# *In situ* biomarker discovery and label-free molecular histopathological diagnosis of lung cancer by ambient mass spectrometry imaging

**DOI:** 10.1038/srep14089

**Published:** 2015-09-25

**Authors:** Tiegang Li, Jiuming He, Xinxin Mao, Ying Bi, Zhigang Luo, Chengan Guo, Fei Tang, Xin Xu, Xiaohao Wang, Mingrong Wang, Jie Chen, Zeper Abliz

**Affiliations:** 1 State Key Laboratory of Bioactive Substance and Function of Natural Medicines, Institute of Materia Medica, Chinese Academy of Medical Sciences and Peking Union Medical College, Beijing 100050, P. R. China; 2 Department of Pathology, Peking Union Medical College Hospital, Chinese Academy of Medical Sciences and Peking Union Medical College, Beijing 100730, P. R. China; 3 State Key Laboratory of Precision Measurement Technology and Instruments, Department of Precision Instruments, Tsinghua University, Beijing 100084, P. R. China; 4 State Key Laboratory of Molecular Oncology, Cancer Institute, Chinese Academy of Medical Sciences and Peking Union Medical College, Beijing 100021, P. R. China

## Abstract

Sensitive and spatial exploration of the metabolism of tumors at the metabolome level is highly challenging. In this study, we developed an *in situ* metabolomics method based on ambient mass spectrometry imaging using air flow-assisted desorption electrospray ionization (AFADESI), which can spatially explore the alteration of global metabolites in tissues with high sensitivity. Using this method, we discovered potential histopathological diagnosis biomarkers (including lipids, amino acids, choline, peptides, and carnitine) from 52 postoperative lung cancer tissue samples and then subsequently used these biomarkers to generate images for rapid and label-free histopathological diagnosis. These biomarkers were validated with a sensitivity and a specificity of 93.5% and 100%, respectively. Moreover, a single imaging analysis of a cryosection that visualized all these biomarkers, taking tens of minutes, revealed the type and subtype of the cancer. This method could potentially be used as a molecular pathological tool for rapid clinical lung cancer diagnosis and immediate image-guided surgery.

Molecular pathology testing is becoming an integral part of clinical practice by pathologists^1^,^2^. However, the current methods for molecular pathology diagnosis, including immunohistochemical analysis (IHC), fluorescence *in situ* hybridization (FISH), and polymerase chain reaction (PCR), are focused primarily on monitoring changes in macromolecules (i.e., mRNAs and proteins) in a low-throughput manner^3^. Moreover, most of these molecular pathology techniques are complex and time-consuming owing to their requirements for special antibodies or chemical labeling^4^. Currently, surgical excision remains the main curative therapeutic modality for cancer^5^; intraoperative consultation between a pathologist and a surgeon requires rapid and accurate diagnosis to guide immediate surgical management^6^,^7^.

Metabolites serve as direct signatures of biochemical activity and are therefore easier to correlate with phenotype^7^. Several reports have demonstrated that comprehensive metabolite measurement and evaluation through metabolomic profiling enable more thorough characterization of pathological conditions^8^,^9^,^10^,^11^,^12^. Metabolomics—the study of the entire set of small-molecule metabolites present in a biological sample—is widely used to identify and evaluate potential diagnostic biomarkers in a variety of malignancies^13^,^14^. Because tissues are the locations of most cancerous lesions, tissue metabolite profiling is a powerful tool for deciphering the abnormal metabolisms of tumors^15^,^16^. Although mass spectrometry-based metabolomics methods are widely used, they require multi-step metabolite extraction and separation; consequently, a loss of pathophysiology-associated localization information in tissue samples represents a major limitation on their utility.

Mass spectrometry imaging (MSI), a spatially resolved label-free bioanalytical technique, can directly identify and map the spatial arrangement of known or unknown molecular species in tissues in association with histological features; hence, this approach can be considered a high-throughput and label-free form of IHC^17^,^18^,^19^. Matrix-assisted laser desorption ionization (MALDI) MSI allows direct analysis of the spatial distribution of proteins in tissue specimens, enabling the discovery of diagnostic and prognostic markers of cancer^20^,^21^. Desorption electrospray ionization (DESI) MSI, one of the earliest ambient MSI methods, has been applied to the classification of tumors and can provide valuable prognostic information such as tumor subtype and grade, mainly in terms of the lipid profile^22^,^23^.

Lung cancer is by far the most common cancer and the largest contributor to cancer-related mortality throughout the world^24^. Several groups have demonstrated that on-tissue digestion combined with MALDI MSI can be used to histopathologically study lung cancer via interrogation of the protein expression patterns in tissues^25^,^26^,^27^.

We recently developed a novel air flow-assisted desorption electrospray ionization (AFADESI) that uses high-rate air flow to enhance ion transmission and ionization efficiency under ambient conditions. Using this technique, we successfully achieved rapid real-time monitoring^28^ and whole-body molecular imaging of a known anticancer agent^29^. Furthermore, we investigated the molecular mechanism of drug action *in vivo*^30^.

In the current study, we developed an *in situ* metabolomics method based on the ambient AFADESI-MSI technique and multivariate statistical analysis (MVSA), with the aim of simultaneously exploring the spatial distribution of all unknown metabolites under a pathological state within a tissue sample. The goal was to address the challenges outlined above regarding a rapid and direct pathological diagnosis of cancer. The strategy for the development of this method is shown in Fig. 1. AFADESI-MSI was used to acquire a massive amount of data associated with multiple species of endogenous metabolites from different pathological conformation tissues, while preserving *in situ* and spatial information. Then, the MVSA method was conducted so as to screen and identify reliable biomarker candidates from low-content metabolites. Subsequently, MSI on cryosections generated images with these biomarkers for rapid, label-free histopathological diagnosis. Finally, when the *in situ* metabolomics method based on AFADESI-MSI was applied to the histological diagnosis of lung cancer, the results demonstrated that this method is effective and feasible.

## Results

### Global metabolite profiling in lung cancer tissues

To reveal alterations in global metabolites associated with cancer, it is essential to use a method that allows evaluation of complex tissues with high throughput and sensitivity. To this end, we constructed an AFADESI-MSI platform in-house and used it to image metabolites in tissue. Because it uses high-speed air flow, this technology can achieve remote ambient MS analysis with high sensitivity, particularly in surface analyses of large objects^28^, which has potential advantages in the context of rapid detection of clinical materials. Using this system, we analyzed 52 clinical postoperative lung tissues, including adenocarcinoma (AC), squamous cell lung carcinoma (SCC), and corresponding adjacent normal tissues (for information on the samples, see in Supplementary Table S1), with AFADESI-MSI in both the positive- and negative-ion modes. In general, the scanning time for the AFADESI-MSI analysis of a tissue section with an area of 1 × 1 cm^2^ was approximately 40 minutes. Representative AFADESI-MS spectra from several histological types of lung cancer tissue are shown in Supplementary Fig. S1. An abundance of peaks, corresponding to all types of metabolites, was observed in the AFADESI-MS spectra. Most of the detected metabolites were tentatively assigned according to accurate mass measurements, MS/MS analyses, and database searches^22^,^31^. The primary ions observed in the low mass/charge (*m*/*z*) range corresponded to amino acids, choline, peptides, and carnitine, among others, whereas above *m/z* 500, the spectra were dominated by various lipids, primarily sphingomyelins (SMs), glycerophosphoethanolamines (PEs), glycerophosphocholines (PCs), phosphatidylserines (PSs), and lysophosphatidylcholines (LysoPCs). These results suggested that AFADESI-MSI has the ability to acquire multiple types of *in situ* information about endogenous metabolites from tissues, with high sensitivity and broad metabolite coverage.

### Multivariate statistical analyses

Although different types of lung tissues have different metabolite profiles, it would be inappropriate to discriminate among them based solely on a single mass spectrum. Compared with other biological samples, such as plasma and urine, tissue is not a uniform biological sample. Therefore, sampling of different regions will yield different metabolite profiling due to the differentiated tissue morphology and tissue type and even the number of tumor cells. Furthermore, such an approach would not be practical in clinical histopathological applications because single spectra cannot represent the common features of whole samples or reflect intra-sample variability. Therefore, we propose an *in situ* metabolomics method based on AFADESI-MSI that integrates histology-defined features extraction (HDFE) from ROIs and multivariate statistical analysis (MVSA) to investigate changes in metabolites and screen for potential diagnostic biomarkers in massive MSI datasets.

HDFE from ROIs was based on the visual ion image and the corresponding optical image of the tissue section. Supplementary Fig. S2. shows how histopathological features were extracted from the raw MSI datasets. Specifically, an ROI was defined on the basis of examination of the hematoxylin and eosin (H&E)-stained adjacent section (optical image), and the resultant outline was then drawn over the ion image. The profiles of metabolites in specific regions were extracted from the ROI, resulting in a two-dimensional data matrix (*m/z*, intensity) that contained histopathological information. All the separated sample matrixes were then grouped according to histopathology after peak alignment, normalization, and background deduction, and then subjected to further MVSA.

**Figure 2:**
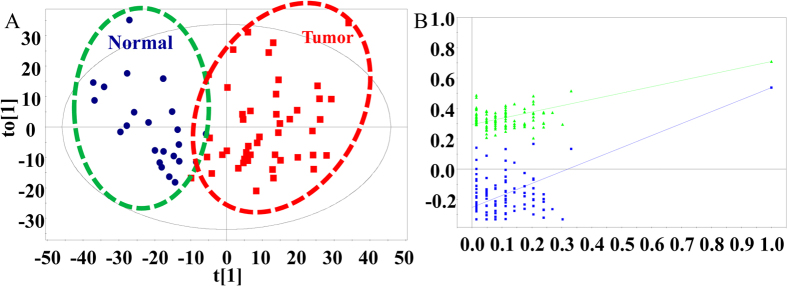
(**A**) Score plot of the OPLS-DA models (R^2^(Y) = 71.0% and Q^2^ = 57.3%) derived from AFADESI-MSI data to differentiate lung cancer tissue (Red squares) and adjacent normal tissue (Blue circles). Sample points for different histopathological classes are grouped and clearly separated from one another. (**B**) Permutation test results (after 100 permutations) of the PLS-DA models (R^2^ = 0.294, Q^2^ = −0.253) correspond to the OPLS-DA models, indicating that the model was not overfitted and can be further processed.

We used a supervised statistical analytical method—orthogonal projections to latent structures discriminant analysis (OPLS-DA)—to describe the disease state of tissue and screen potential diagnostic biomarkers^32^. As shown in Fig. 2, the score plot exhibited a clear separation between malignant and adjacent normal tissues, suggesting that the metabolites were significantly altered in the tumor tissue. The classification of the tumor and non-tumor groups resulted in one predictive (t_p_) and two orthogonal (t_o_) (1 + 2) components with a cross-validated predictive ability Q^2^ (cum) of 57.3%. In addition, a value of 52.7% variance in X [R^2^(X)] was used to account for 71.0% of the variance of Y [R^2^(Y)]. To further validate this model, we performed a random permutation test with the partial least squares discriminant analysis (PLS-DA) model, corresponding to the OPLS-DA model across three components. Validation with 100 random permutation tests generated intercepts R^2^ = 0.294 and Q^2^ = −0.253, indicating that the model was not overfitted^33^,^34^.

### Diagnostic biomarker discovery and evaluation

Following the MVSA analysis, we performed multi-step biomarker screening processes, including S-plot, VIP value, and *t*-test, to discover reliable biomarkers^33^,^35^. The result was identification of 38 variables that discriminated between tumor and non-tumor samples. Furthermore, because the distinct features of an MSI dataset are composed of a large number of points (couple [*m/z*, intensity]) with spatial information, the two-dimensional distribution maps of ions of interest can be intuitively reconstructed for further biomarker screening. After the imaging tests (Supplementary Fig. S3), 12 discriminated variables were excluded because they did not have distinct contours relative to the background area, leaving 26 variables as potential biomarker candidates that reflected metabolic feature differences between tumor and non-tumor tissues. Ultimately, 24 potential biomarkers were tentatively identified. Detailed information is provided in Supplementary Table S2. All of these potential biomarkers were upregulated in lung cancer tissues.

**Figure 3:**
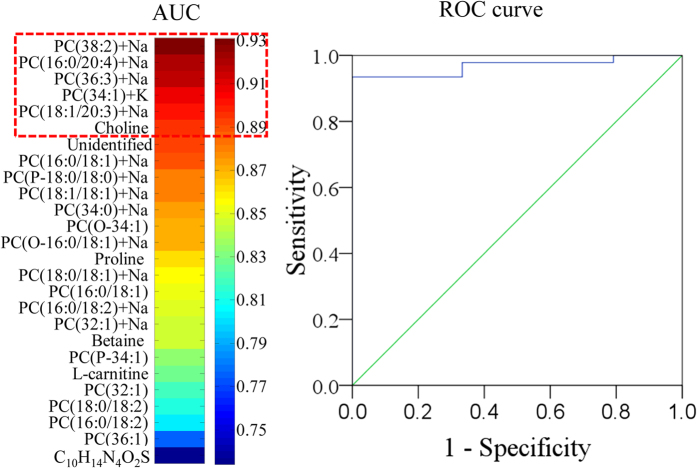
A visualization of the discriminatory power across individual diagnostic biomarker candidates and classification performance of the combined biomarker group. (**A**) A heat map was used to depict the discriminatory power of each metabolite, estimated by the AUC. The colors correspond to the AUC values: red and blue represent high and low values, respectively. (**B**) The ROC curve illustrates the combined discriminatory performance of a group of valuable clinical biomarkers with AUC >0.90.

We next performed in-depth receiver operating characteristic (ROC) analysis to evaluate the reliability of these putative biomarkers. Heat maps were created to illustrate the discriminatory power of each potential biomarker, and the biomarkers were ranked in order of their area under the curve (AUC) values. All the potential biomarkers had AUC values within the range of 0.73–0.93. Metabolites with AUC >0.90, including choline, [PC(38:2) + Na]^+^, [PC(16:0/20:4) + Na]^+^, and [PC(36:3) + Na]^+^, [PC(34:1) + K]^+^, [PC(18:1/20:3) + Na]^+^, were combined into a group of valuable clinical biomarkers that was then used to evaluate discrimination performance. After combining these biomarkers by binary logistic regression, we performed ROC curve determination, which revealed that the valuable clinical biomarker group had an AUC of 0.968, providing the best available discrimination between lung cancer and non-tumor tissue. The sensitivity and specificity, calculated at the best cutoff points, were 93.5% and 100%, respectively (Fig. 3). The results of the ROC analysis further demonstrated that the *in situ* metabolomics method based on AFADESI-MSI, combined with HDFE and MVSA, could effectively identify reliable biomarkers from massive MSI data and eliminate the effects of sample variability.

### Label-free molecular histopathological diagnosis of lung cancer

Clinical management and ultimate prognosis of lung tumors depend largely on tumor type, subtype, and grade. Molecular imaging by MSI allows correlation of the identity of a metabolite with its spatial distribution on the tissue surface; this information has been demonstrated to be important in tumor diagnosis and surgical resection^23^. Hence, after the discovery of potential biomarkers via *in situ* metabolomics with AFADESI-MSI, we further validated the ability of these biomarkers to diagnose lung cancer according to the corresponding ion images.

**Figure 4:**
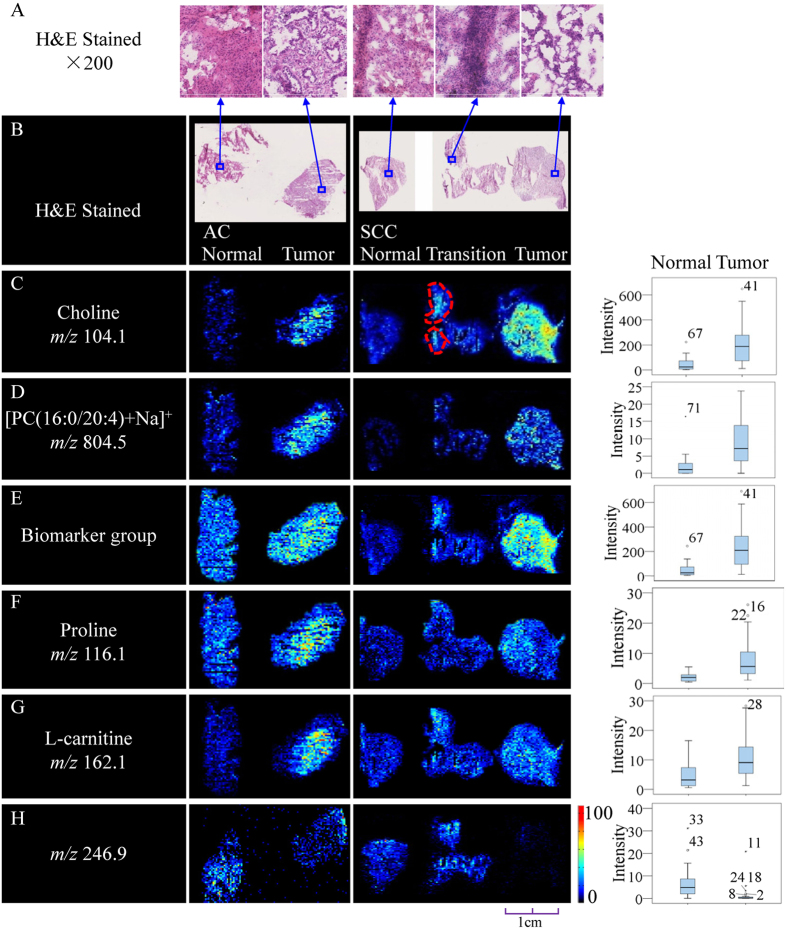
Distributions of representative potential biomarkers (group) across tumors and adjacent normal tissue sections from AC and SCC lung cancer. (**A**,**B**) Optical images of corresponding H&E-stained sections and the amplified figures (×200). (**C**–**H**) Ion images and corresponding statistical box plots. Column 1: potential biomarkers with their molecular ions (m/z); Column 2: comparison of ion images between tumors and adjacent normal tissue sections from AC; Column 3: comparison of ion images between tumors and adjacent normal tissue sections from SCC; Column 4: the statistical box plots show the ion intensity of tumors and adjacent normal tissue sections.

The distribution of potential biomarkers across the tissue was visualized as ion images, shown in Fig. 4 and Supplementary Fig. S3. The representative ion images from SCC and AC tissues delineate the histopathology of lung cancer at the molecular level. The imaging results suggested that the intensities of the signals corresponding to potential biomarkers were elevated in cancerous tissue. Furthermore, the spatial distributions of potential biomarkers presented in the ion images were consistent with the statistical trends shown in the box plots (Fig. 4). Remarkably, close examination of the ion images in the transition region containing normal and cancerous tissue revealed a heterogeneous distribution of metabolites; specifically, the area marked with a red dotted line in Fig. 4C presented with a higher ion signal intensity. Compared with the corresponding H&E stained sections, tumor cells were clearly observed in this area, which is the major factor contributing to this phenomenon. In addition, a discriminated ion at *m/z* 246.9 in the negative-ion mode was identified as a potential diagnostic biomarker. In contrast to other potential biomarkers, this ion (Fig. 4H) exhibited an inverse distribution with high expression in adjacent normal tissue but low concentration in tumor tissue. Therefore, the accuracy of histopathological diagnosis of tumors would be improved by the specific inverse distributions of multiple biomarkers. This result emphasizes that biomarker ion images discovered via *in situ* metabolomics based on AFADESI-MSI not only clearly delineate the tumor margin but also characterize the neoplasms’ infiltration. In addition, these ion images show that the potential biomarkers distribute uniformly in the tissue sections and that tumors display pronounced heterogeneity in many morphological and physiological features; MSI technology thus shows an extraordinary superiority for characterization of tumor tissues.

Lipids have high ionization efficiency because they possess a polar head and are therefore more easily detected than other endogenous metabolites. For this reason, and because they are abundant in tissues, these molecules are key targets for MSI studies^10^,^36^. However, the *in situ* metabolomics method based on AFADESI-MSI also identified many non-lipid endogenous metabolites present at low concentrations, including amino acids and carnitine, as potential biomarkers (Fig. 4F,G). Thus, this strategy can minimize ion suppression from high-abundance metabolites or a complex matrix, at least to some extent, enabling exploration of large numbers of metabolites that are significantly associated with the histopathology of tissues and potentially providing insight into the mechanisms and etiology of disease.

**Figure 5:**
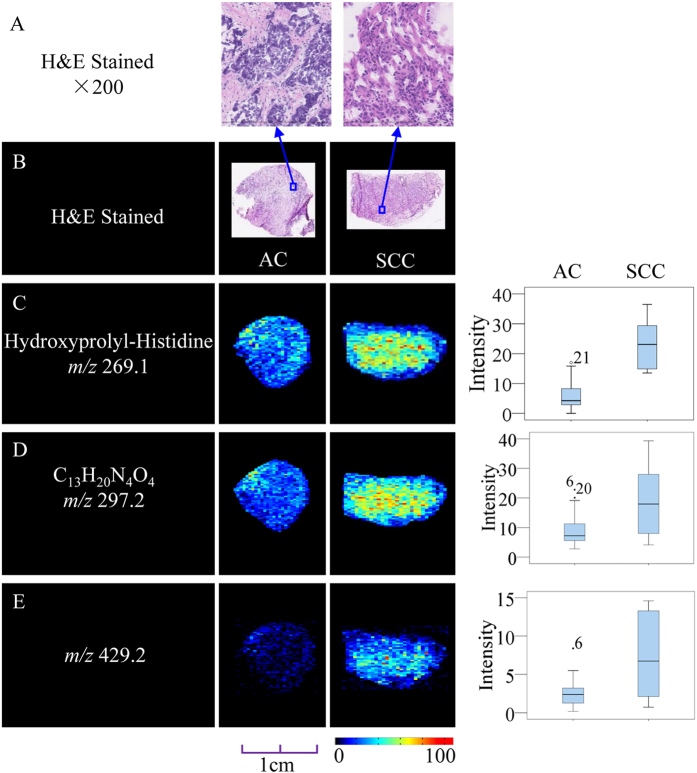
Distribution of representative potential biomarkers across tissue sections from subtypes of lung cancer. (**A**,**B**) Optical images of corresponding H&E-stained sections and the amplified figures (×200). (**C**–**E**) Ion images and corresponding statistical box plots. Column 1: potential biomarkers with their molecular ions (*m/z*); Column 2: ion images from tissue sections of AC; Column 3: ion images from tissue sections of SCC; Column 4: the statistical box plots show the ion intensity of tumor tissue sections with subtypes of AC and SCC.

Because of differences in the origin, treatment, and prognosis of various cancer subtypes, it is essential to discriminate between histological types. Using the *in situ* metabolomics method described above, we investigated the correlation between the tissue metabolite profiles of SCC and AC lung cancer. After an OPLS-DA model was generated, the score plots exhibited a clear discrimination (Supplementary Fig. S4A) between SCC and AC, and three metabolites that accounted for this discrimination were tagged as potential biomarkers. Detailed information is provided in Supplementary Table S3. Representative imaging analysis results (Fig. 5) revealed that the signal intensities of all three metabolites were higher in SCC than in AC; this trend was in accord with the statistical results, represented in the box plots.

In addition to discrimination of pathological type, we investigated the differentiation degree of SCC and AC using these methods. Poorly differentiated cancer cells are more likely to grow quickly and spread, leading to weaker curative effects and less favorable prognoses, whereas moderate-differentiated and well-differentiated cancer cells are prone to be slow-growing and less aggressive. This information is valuable for determining treatment plans and prognosis. Hence, we divided the tumor samples into two groups—“poorly differentiated” and “moderate/well-differentiated”—and then conducted *in situ* metabolomics via AFADESI-MSI, OPLS-DA modeling, and variable screening approaches to discriminate between SCC and AC of different degrees.

**Figure 6:**
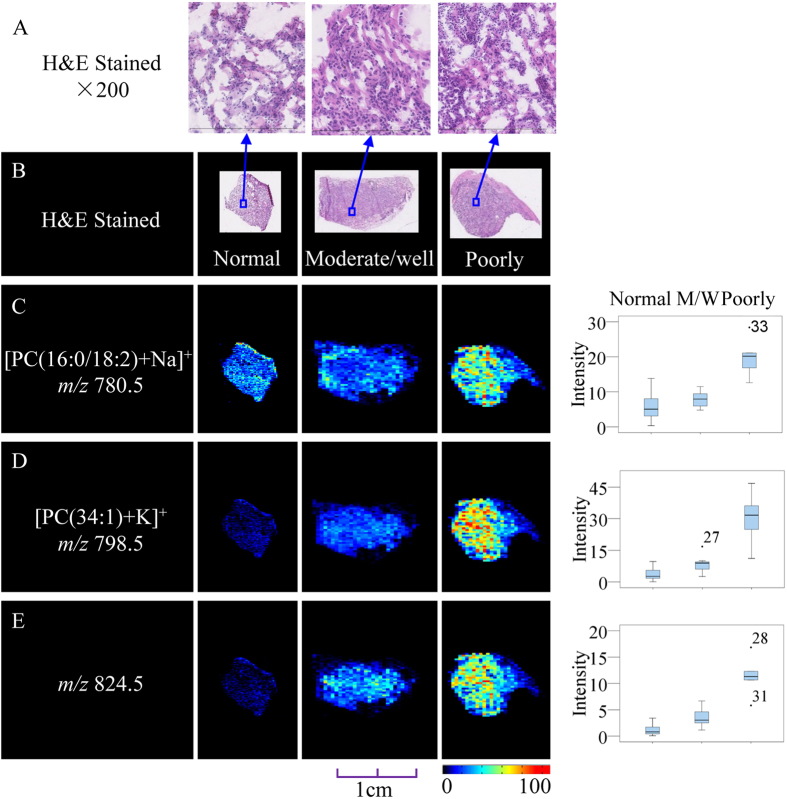
Distribution of representative potential biomarkers across tissue sections from SCC, with degree of differentiation in the tumorous and adjacent normal tissue. (**A**,**B**) Optical images of the corresponding H&E-stained sections and the amplified figures (×200). (**C**–**E**) Ion images and corresponding statistical box plots. M/W: moderate/well. Column 1: potential biomarkers with their molecular ions (m/z); Column 2: ion images from adjacent normal tissue sections; Column 3: ion images from tumorous tissue sections with moderate/well differentiation; Column 4: ion images from tumorous tissue sections with poorly differentiation; Column 5: the statistical box plots show the ion intensity of the tumorous and adjacent normal tissue sections with the degree of differentiation.

In the SCC samples, there was a clear separation (Supplementary Fig. S4B) between the poorly-differentiated and moderate/well-differentiated groups; a number of lipid compounds, such as [PC(16:0/18:2) + Na]^+^ (*m/z* 780.5), [PC(34:1) + K]^+^ (*m/z* 798.5), and *m/z* 824.5 (P < 0.01,VIP >1.5), were all expressed in the adjacent normal, moderate/well and poorly-differentiated groups in increasing order of expression levels (Fig. 6). In the AC samples (Supplementary Fig. S4C), three metabolites that contributed the most discriminatory power in the model were selected as potential biomarkers associated with the differentiation degree of AC. These metabolites are all lipid species, including one sphingomyelin (SM) and two phosphatidylcholines (PC) (Supplementary Table S4). All these species were upregulated in the moderate/well differentiated group relative to that in the poorly-differentiated and adjacent normal groups (Supplementary Fig. S5); this trend is apparent in the representative images and box plots.

## Discussion

Cancer cells grow and divide at an unregulated, quickened pace due to damage or changes to the DNA, and it is well known that cancer cells have atypical metabolic characteristics relative to normal cells^37^. Therefore, cancer cells must generate enough energy and acquire or synthesize biomolecules at a rate sufficient to meet the need for intensified cell membrane synthesis and cell replication^38^. Using *in situ* metabolomics based on AFADESI-MSI, we found that 20 specific lipid species were significantly elevated in tumor tissues; these additional lipids are likely to be used by tumor cells for the synthesis of cell membranes, lipid rafts, and lipid-modified signaling molecules^39^. Choline itself plays a critical role in the structure and function of biological membranes in all cells, and numerous studies have demonstrated that elevation of the choline level in cancer cells may correlate with a higher rate of choline transport due to increased expression of choline-transporting transmembrane systems^40^. In addition, we detected elevated levels of proline, betaine, and L-carnitine in malignant tumors. Proline is an α-amino acid, and a previous study demonstrated that the oncogenic transcription factor c-MYC (MYC) can markedly increase the biosynthesis of glutamine-derived proline^41^,^42^. In another report, metabolomic analysis of serum and tissue samples from lung cancer patients revealed that proline was elevated in both serum and tumor tissue, which suggests that when a tissue becomes cancerous, metabolites move from the blood into the tissue to promote cancer cell proliferation^43^. Betaine is a major methyl donor that can be synthesized from choline oxidation. The upregulation of betaine in lung cancer may be due to enhanced choline transport 44,45. Carnitine plays an important role in energy metabolism by mediating the transport of long-chain fatty acids across the inner mitochondrial membrane^46^,^47^; the high energy expenditure in cancer cells may perturb carnitine homeostasis, resulting in high overall levels of this compound^45^.

The three molecules were capable of discriminating the pathological types of cancer, indicating that tumors with different pathological features possess different metabolite profiles. The differences in metabolites in histological subtypes of lung cancer may represent the influence of cancer as well as inflammation^43^. In addition, the relationship between metabolites and the degree of cell differentiation in lung cancer indicates that lipid compounds, especially PC, play a vital role in tumor differentiation. However, the opposing trends in the levels of potential biomarkers in AC and SCC remain to be investigated.

We developed an *in situ* metabolomics method based on ambient MSI and demonstrated that it could effectively discover reliable biomarkers, with spatial information, for use in histological diagnosis. This method takes advantage of the capacity of AFADESI-MSI to explore the diversity of global and *in situ* metabolite information from tissue in a single experiment. The detection coverage for endogenous metabolites was greatly extended by adopting this method, specifically by including not only lipids but also amino acids, choline, peptides and carnitine. Furthermore, the histology-defined data-extraction method allowed acquisition of histopathological information from ROIs, and MVSA enabled identification of diagnostic biomarkers from massive MSI data and effectively eliminated the effect of sample variability. As shown by the distributions of these potential biomarkers, this integrated MSI strategy can achieve rapid and direct histopathological diagnosis of cancer.

We used this *in situ* metabolomics method to discriminate between cancerous and adjacent normal tissue, as well as to display the distribution of tumor cells in lung cancer tissue, and thereby identified multiple types of endogenous metabolites as potential biomarkers. The spatial distribution of these potential biomarkers revealed that they were present at significantly different concentrations in lung cancer and its subtypes and were therefore able to distinguish between lung tumor and adjacent normal tissues and to characterize the degree of differentiation and the pathological type. These potential biomarkers were found for the first time using the mass spectrometry imaging method; except for *m/z* 255.0910 (C_10_H_14_N_4_O_2_S), other potential biomarkers have been previously identified using other technologies, such as NMR and LC-MS, from serum and/or urine. However, analysis of additional unknown samples should be performed on a large scale to further validate the potential biomarkers described in this research. We also applied this method to the histopathological diagnosis of breast and thyroid cancer, and it successfully distinguished the tumor and adjacent normal tissues as shown in images of discriminated endogenous metabolites (unpublished results). Metabolite-based histopathology provides intuitive molecular phenotypes and biochemical information, breaking through the limitations of traditional H&E and immunohistochemistry methods. Thus, this approach is potentially valuable in the elucidation of pathogenic mechanisms, screening for biomarkers, prediction of therapeutic effect, and identification of new therapeutic targets.

Although this *in situ* metabolomics method based on AFADESI-MSI can perform an untargeted analysis of all the small-molecule metabolites in tissue, there are still many problems that need to be solved. For example, how can we detect more species of endogenous metabolites with low-content in tissue without losing spatial resolution, how can we investigate ion suppression from different pathological conformations, and how can we correctly identify the structure of these diagnostic biomarkers? Nevertheless, direct tissue analysis by the *in situ* metabolomics method based on AFADESI-MSI could be correlated with histological and clinical features for the purpose of disease diagnosis. By providing visual images of biomarker distribution, this technique enables investigation and spatial localization of both known and unknown endogenous metabolites in a single experiment, without the need for labeling. Thus, this method has great potential to contribute to the next generation of metabolite-level diagnostic tests, which will improve both clinical prognoses and patient quality of life.

Based on biomarker discovery and histopathology of specific metabolites, AFADESI-MSI enables accurate delineation of the tumor margin, potentially facilitating optimal tumor resections in the clinic while avoiding recurrence. Furthermore, *in situ* metabolomics combined with fine-needle aspiration (FNA) technology could lead to less invasive early tumor screening and diagnosis in clinical practice.

## Methods

### Sample collection and processing

All post-operative tissue samples, including 52 lung tumor tissues and 21 adjacent normal tissues, were collected in the Peking Union Medical College Hospital and Cancer Institute Hospital, Chinese Academy of Medical Sciences. The study protocols were approved by the Ethics Review Committee of the Peking Union Medical College Hospital, and all the experiments were carried out in accordance with the approved guidelines. Meanwhile, all the patients involved in the study signed the informed consent form and agreed to participate the research. Supplementary Table S1 summarizes the clinical pathological profiles of the lung tumor samples. The samples were snap-frozen in liquid nitrogen and stored at −80 °C before subsequent processing. The frozen samples were cut into 8 μm sections at −20 °C on a cryomicrotome (CM 3050S, Leica Microsystems, Wetzlar, Germany) and thaw-mounted onto microscope glass slides (Superfrost Plus slides, Thermo Fisher Scientific, USA). One set of adjacent cryosections was acetone-fixed and subsequently stained using H&E for pathological examination. The sections were stored at −80 °C until they were analyzed. Prior to analysis, the sections were allowed to return to room temperature and then dried under a vacuum in a desiccator for approximately 30 minutes^22^,^48^.

### AFADESI-MSI experimental configuration and MSI analysis

AFADESI-MS imaging analysis of the lung cancer tissue was conducted in both the positive- and negative-ion modes on a commercial Q-TOF mass spectrometer (QSTAR Elite^®^, AB Sciex, USA) equipped with a custom-made AFADESI ion source as well as a computer-controlled imaging stage. Real-time internal mass calibration (using background ions *m/z* 149.0233 and 391.2843) was performed to acquire accurate mass spectra in each MSI experiment. (A more detailed description of the configuration of the AFADESI ion source was provided in previous papers^28^,^29^.) The distance between the transport tube and the curtain plate was 20 mm. The spray voltage was set at (±) 4600 V, and the transport tube voltage was the same as the voltage applied to the curtain plate, i.e., (±) 1200 V. The declustering potential was set at (±) 50 V. The nebulizing gas and curtain gas pressure were set at 60 arb and 20 arb, respectively. The extracting air flow rate was 45 L min^−1^, and the solvent flow rate was 5 μL min^−1^. In the positive-ion scan mode, the spray solvent for the MS acquisition was prepared by mixing methanol and water (9:1, v/v) with 0.1% formic acid, whereas acetonitrile:water (8:2, v/v) was used as the spray solvent in the negative-ion scan mode. The molecular imaging experiments were performed by continuously scanning the tissue surface in the y-direction at a constant velocity of 200 μm s^−1^, separated by a 200 μm vertical step in the x-direction, until the entire tissue sample was assayed^49^.

### Histology-defined features extraction

The raw data (.wiff) collected in each line scan as a separate file containing the endogenous metabolite information for the specimens were converted into the required format (.mat) for image generation. Next, a spatially accurate image depicting the contours of the tissue sections and the spatial distributions of specific ions was plotted using the IMS V 2.5.1 imaging software, developed in-house, with a preselected ion. The same rainbow color scale is used throughout the figures: the red pixels represent the highest signal intensity (100%) of a particular ion; the black pixels represent the lowest signal (0%)^50^.

Based on the visual ion image of a tissue, the software was able to extract spectral data from ROIs. Specifically, an ROI was created by drawing an outline over the ion image based on the pathological characteristics of the H&E-stained adjacent section; thus, the profile of metabolites concerning a particular region could be extracted from the ROI. Moreover, a two-dimensional data matrix (*m/z*, intensity) in .txt format was built up for each ROI. The two-dimensional data matrix from the ROI was averaged in each pixel and adopted for further data analysis to balance the weight of each specimen.

The separated sample dataset matrixes were then imported into the Markerview^TM^ software 1.2.1 (AB SCIEX) for background deduction, peak picking and peak alignment. Specifically, first, an exclusion list of background ions was generated from the background region (process spectra options: for the scan range from 100 to 500 Da, the mass tolerance was 0.03 Da and the minimum required response was 50 Cps, whereas for the mass range from 500 to 1000 Da, the mass tolerance was 0.05 Da and the minimum required response was 1 Cps). When importing the sample dataset matrixes for peak picking, the background ions in the exclusion list were deducted. The list of sample ions was generated with the following parameters: for the scan range from 100 to 500 Da, the minimum required response was 20 Cps, whereas for the mass range from 500 to 1000 Da, the minimum required response was 0.5 Cps. Meanwhile, the ions marked with an isotope checkmark or their intensity at a 0 value account for more than 70% of the samples and were deleted from the peak aligned list. Next, the list was exported in .txt format for later multivariate statistical analysis.

### Multivariate statistical analysis and biomarker screening

The datasets from the ROIs were imported into SIMCA-P 11.0 (Umetrics AB, Umea, Sweden), centered, and Pareto scaled to reduce the impact of noise and artifacts on the models. The significantly changed metabolites that contributed most to the model were selected as potential biomarkers for further study. More specifically, an S-plot showing the covariance and correlation between the metabolite variables and the model was used to select the discriminating variables. Variables with both high covariance and high correlation were preferentially selected. The VIP value reflects the influence of every variable on the classification; only variables with a VIP value above 1.5 were considered. Moreover, a jack-knifing confidence interval was applied, and the metabolites with a positive score were kept as further candidates^33^,^35^. The biomarker candidates were further confirmed by an independent *t*-test (P < 0.01) using PASW Statistics 18.0 (formerly SPSS Inc., Chicago, IL USA), and the variation and comparison in the levels of the potential biomarkers between the experimental groups were presented in box plots. Finally, retrospective imaging tests were performed to further confirm the reliable potential biomarkers, according to whether their ion images have distinct contours relative to the shape of the tissue section.

### Metabolite identification

Metabolite assignments were tentatively confirmed by tandem mass spectrometry (MS/MS) experiments, using the LC-MS technique and AFADESI-MS. For the LC-MS/MS experiment, the frozen lung tissue specimens were weighed at approximately 50 mg prior to homogenization in an ice-cold mixture solution (methanol 410 μL and water 210 μL); then, 280 μL of methylene dichloride and 210 μL of water were added. The samples were thoroughly vortexed for 2 minutes, followed by centrifugation at 15000 rpm for 10 min at 4 °C. The resulting supernatants that were methanol and water were collected and dried under nitrogen. The dried extracts were stored at −80 °C until the LC-MS/MS analysis. Before the analysis, the extracts were resuspended in 1200 μL of acetonitrile/water (8:2). The analyses were performed using an UltiMate 3000 RSLC System (Dionex, Thermofisher, USA) coupled to a Q-Orbitrap mass spectrometer (Q-Exactive, Thermofisher, USA). The MS spray voltages were 3.5 kV in the positive mode. The capillary temperature was set at 350 °C with the sheath gas at 40 arbitrary units and the aux gas at 11 arbitrary units. The chrom. peak width (FWH) was 15 s, and the mass scan range was set from 70 to 1000 Da. The resolution of the Orbitrap was set at 70,000. The MS/MS data were collected with the collision energy between 10 and 45 eV. A linear 30 min water (5 mM ammonium acetate) to acetonitrile gradient was run on a Phenomenex Kinetex HILIC column (2.6 μm 2.1 × 150 mm) in the positive electrospray mode. The AFADESI-MS/MS analysis was performed directly on the tissue samples, with a series of line scans across the tissue sample until the desired results were achieved, and the detailed scan parameters were shown in AFADESI-MSI experimental configuration and MSI analysis; the collision energy was set between 10 and 35 eV. Comparison with literature data and authentic standards, as well as exact molecular weights (accurate mass of molecular and product ions) and a query of the LIPID MAPS (http://www.lipidmaps.org/), Massbank (http://www.massbank.jp/), HMDB (http://hmdb.ca/), and METLIN (http://metlin.scripps.edu)^49^,^52^ databases, resulted in tentative assignment of the structures of these potential biomarkers.

### Intra-day reproducibility

To ensure data stability and reliability, intra-day reproducibility was investigated by scanning red bars (Rhodamine B solution, *m/z* 443.2) on 6 successive days under the same experimental conditions. The relative standard deviation (RSD) values of the average peak area on 6 successive days were 18.19%. For biomarker studies, the FDA guidelines specify an RSD <20% as an acceptable level of precision^52^.

### Data quality assessment

When multiple analytical experiments are to be performed, the stability and suitability of the analytical system must be taken into consideration. In this study, the dataset extracted from the background area was recognized as the QC (quality control) samples, applied to assess and ensure the reliability of the analytical processes. According to a previous report, the most straightforward way to begin analysis of the QC data is to use principal component analysis (PCA), an unsupervised technique^53^. Multivariate analysis results of the QC samples are shown in Supplementary Fig. S6. The dataset deviation was <2 SD, suggesting that the AFADESI-MSI data are worthy of further study.

## Additional Information

**How to cite this article**: Li, T. *et al.* *In situ* biomarker discovery and label-free molecular histopathological diagnosis of lung cancer by ambient mass spectrometry imaging. *Sci. Rep.* **5**, 14089; doi: 10.1038/srep14089 (2015).

## Supplementary Material

Supplementary Information

Supplementary Data file S1

## Figures and Tables

**Figure 1:**
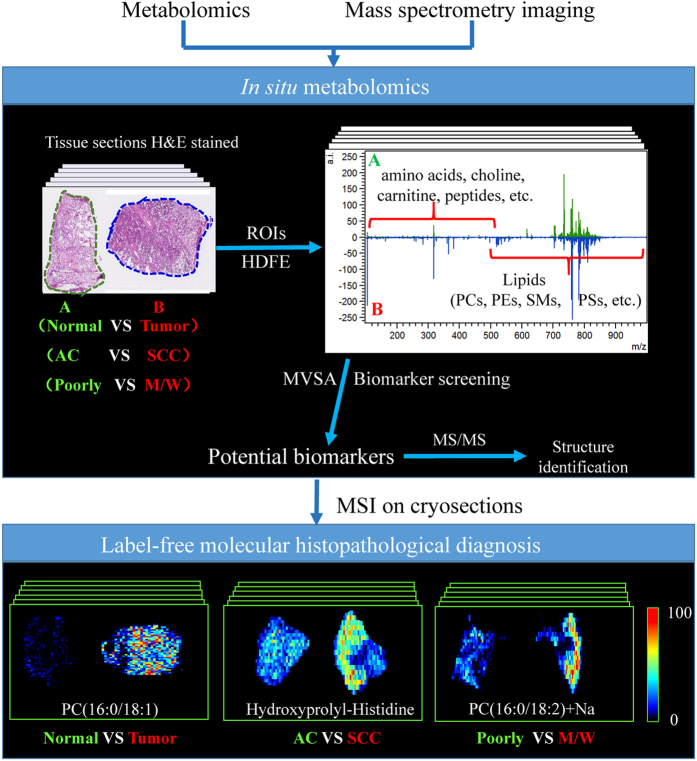
The strategy for developing an *in situ* metabolomics method to discover diagnostic biomarkers for a rapid, direct and label-free molecular histopathological diagnosis of cancer. (ROIs: regions of interest; HDFE: histology-defined features extraction; MVSA: multivariate statistical analysis, AC: adenocarcinoma, SCC: squamous cell carcinoma; Poorly: poorly differentiated lung cancer; M/W: moderate/well-differentiated lung cancer)
