# Supplementary material for: In situ biomarker discovery and label-free molecular histopathological diagnosis of lung cancer by ambient mass spectrometry imaging
Source: Sci Rep. 2015 Sep 25;5:14089. doi: 10.1038/srep14089 (PMC4585892; doi:10.1038/srep14089)
Supplement: Supplementary Information [file srep14089-s1.doc]

Title: *In situ* biomarker discovery and label-free molecular histopathological diagnosis of lung cancer by ambient mass spectrometry imaging

**Authors:** Tiegang Li^1^*^+^*, Jiuming He^1^*^+^*, Xinxin Mao^2^, Ying Bi^3^, Zhigang Luo^1^, Chengan Guo^3^, Fei Tang^3^, Xin Xu^4^, Xiaohao Wang^3^, Mingrong Wang^4^, Jie Chen^2^*, Zeper Abliz^1^*

**Affiliations:**

^1^State Key Laboratory of Bioactive Substance and Function of Natural Medicines, Institute of Materia Medica, Chinese Academy of Medical Sciences and Peking Union Medical College, Beijing 100050, P. R. China

^2^Department of Pathology, Peking Union Medical College Hospital, Chinese Academy of Medical Sciences and Peking Union Medical College, Beijing 100730, P. R. China

^3^State Key Laboratory of Precision Measurement Technology and Instruments, Department of Precision Instruments, Tsinghua University, Beijing 100084, P. R. China

^4^State Key Laboratory of Molecular Oncology, Cancer Institute, Chinese Academy of Medical Sciences and Peking Union Medical College, Beijing 100021, P. R. China

*Corresponding author: Prof. Dr. Zeper Abliz and Prof. Dr. Jie Chen

Address: No. 1 Xiannongtan street, Xicheng District, Bejing 100050, P. R. China, Email: zeper@imm.ac.cn (Z. A.)

Address: No. 1 Shuaifu Garden street, Dongcheng District, Beijing 100730, P. R. China, Email: xhblk@163.com (J. C.)

+These authors contributed equally to this work.

**Supplementary Information**

1. Table S1. Histopathological characteristics of the postoperative lung cancer tissues included in this study.
2. Fig. S1. Mass spectra from human lung cancerous tissue and adjacent normal tissue, acquired by AFADESI-MSI in positive-ion mode without labeling.
3. Fig. S2. Definition of regions of interests (ROIs) and corresponding histopathological features extraction from raw MSI datasets.
4. Fig. S3. Imaging test results of the 38 discriminated variables.
5. Table S2. Potential biomarkers of lung tumors and their tentative identiﬁcations in positive-ion mode.
6. Fig. S4. OPLS-DA models for discrimination of subtype and differentiation degree of lung cancer.
7. Table S3. Potential biomarkers that discriminate SCC and AC, with their tentative assignment results.
8. Table S4. Potential biomarkers that discriminate differentiation degree for AC, with their tentative assignment results.
9. Fig. S5. Distribution of representative potential biomarkers across tissue sections from AC, with degree of differentiation in the tumorous and normal tissue.
10. Fig. S6. Line plots of the data extracted from a blank area generated by PCA using component 1 and 2 shows the stability of AFAI-MSI system.
11. Fig. S7. MS/MS spectra and the proposed dissociation pathways for the discriminated metabolites identification (Tumor VS non-tumor).
12. Fig. S8. MS/MS spectra and the proposed dissociation pathways for the discriminated metabolites identification (AC VS SCC).
13. Fig. S9. MS/MS spectra and the proposed dissociation pathways for the discriminated metabolites identification (Differentiation degree for AC).
14. Data file S1. Processed data for multivariate statistical analyses are available in appendix S1.

**Table S1.** Histopathological characteristics of the postoperative lung cancer tissues included in this study.

| Characteristics | | Adenocarcinoma  (AC) | Squamous cell carcinoma  (SCC) | Total |
| --- | --- | --- | --- | --- |
| Sex | Male | 22 | 15 | 37 |
|  | Female | 15 | 0 | 15 |
|  | Total | 37 | 15 | 52 |
| Histopathological grading | Well-differentiated | 10 | 4 | 14 |
|  | Moderate-differentiated | 13 | 4 | 17 |
|  | Poorly differentiated | 14 | 7 | 21 |
|  | Total | 37 | 15 | 52 |

**
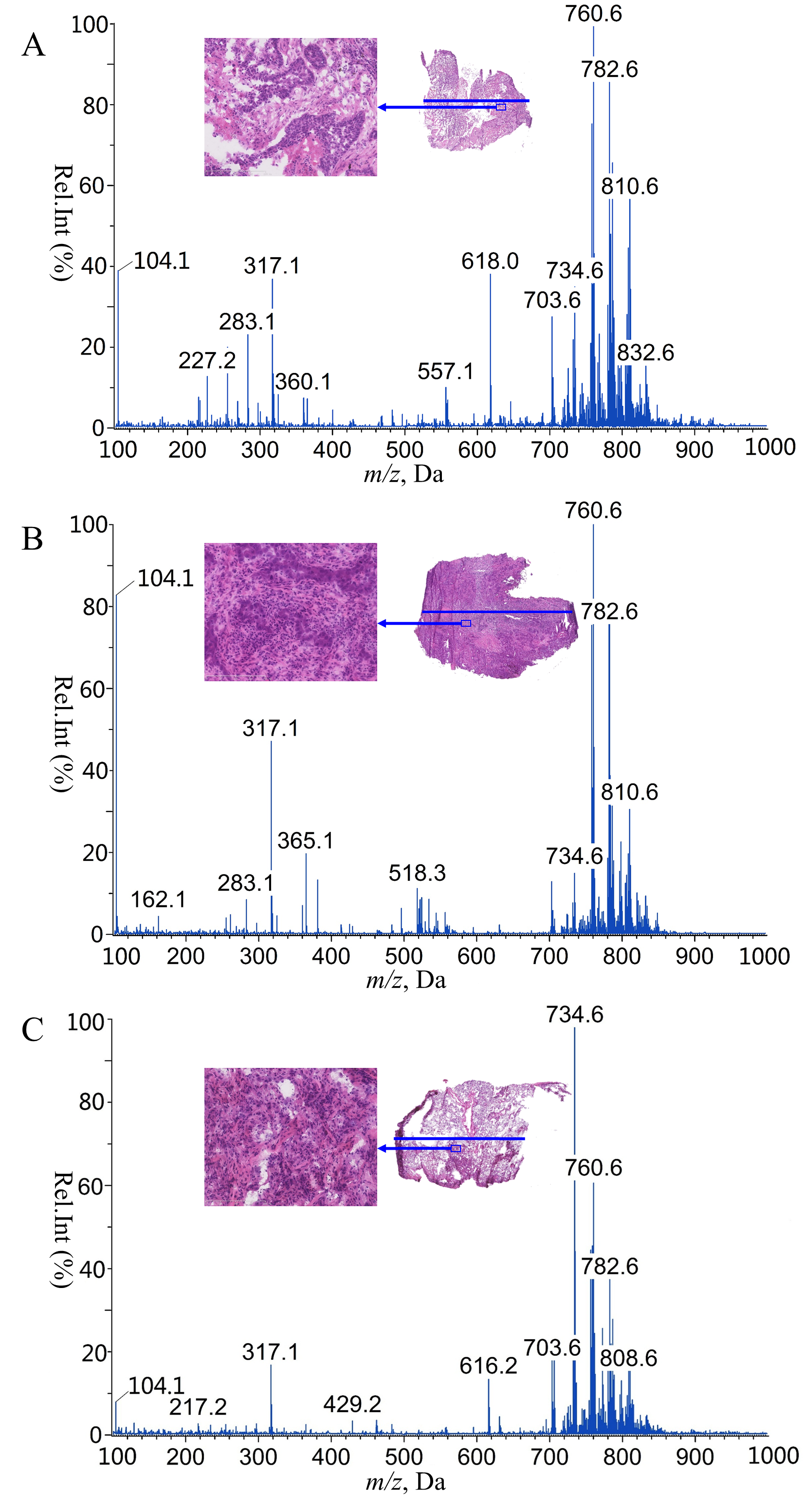
**

**Figure S1.** Mass spectra from human lung cancerous tissue and adjacent normal tissue, acquired by AFADESI-MSI in positive-ion mode without labeling. A) SCC (Poorly differentiated), B) AC (Moderate differentiated) as well as C) Adjacent normal tissue. The insets display the corresponding H&E-stained sections and the amplified figures (×200) showing different histopathological classes of lung tissues. The horizontal lines shown in each corresponding H&E stained tissue sections stands for the region where the mass spectra were acquired from.


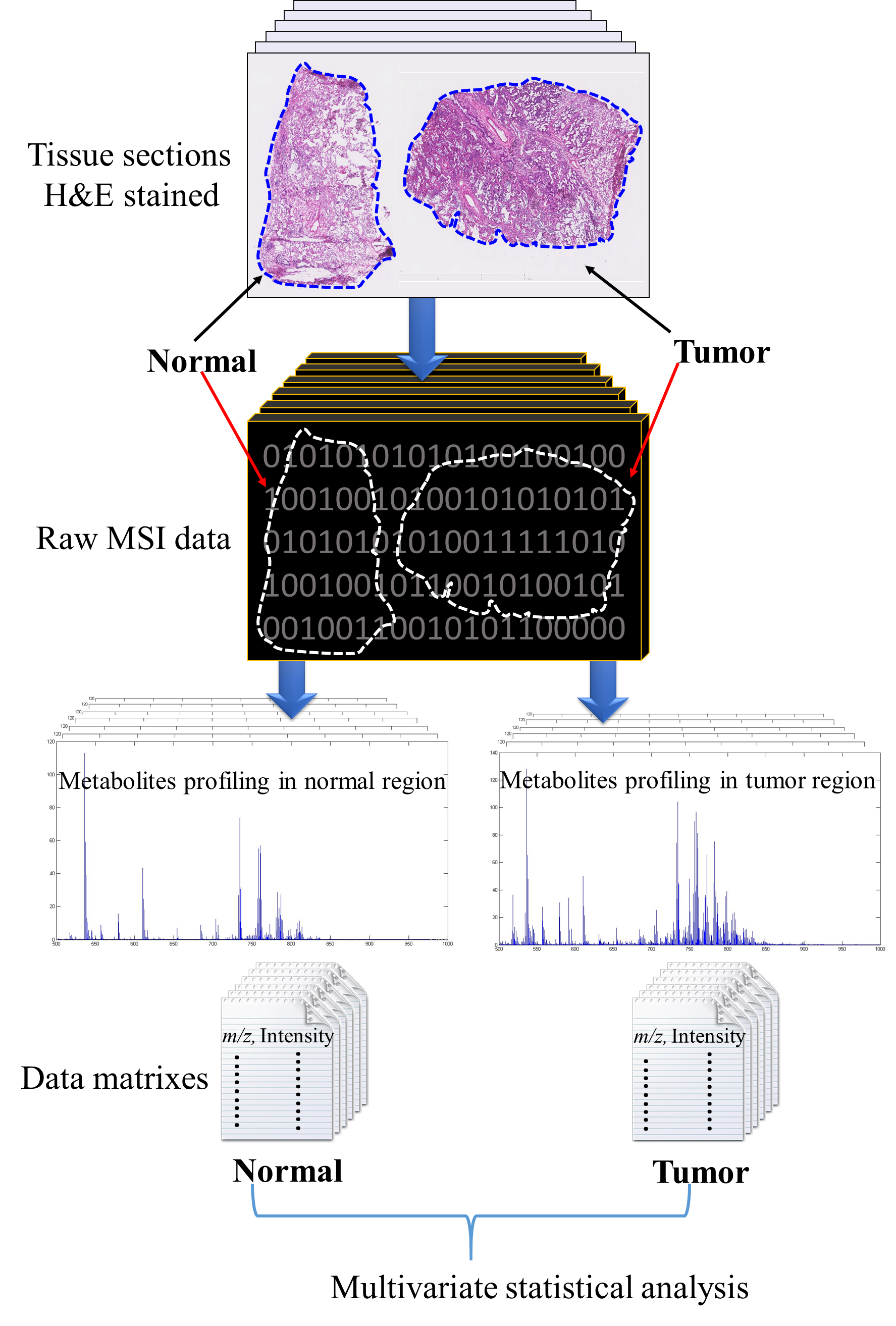


**Figure S2.** Definition of regions of interest (ROIs) and corresponding histopathological features extraction from raw MSI datasets. A homemade software was used to extract the metabolites profile (MS data matrixes, containing *m/z*, intensity and position information) corresponding to histopathological feature.


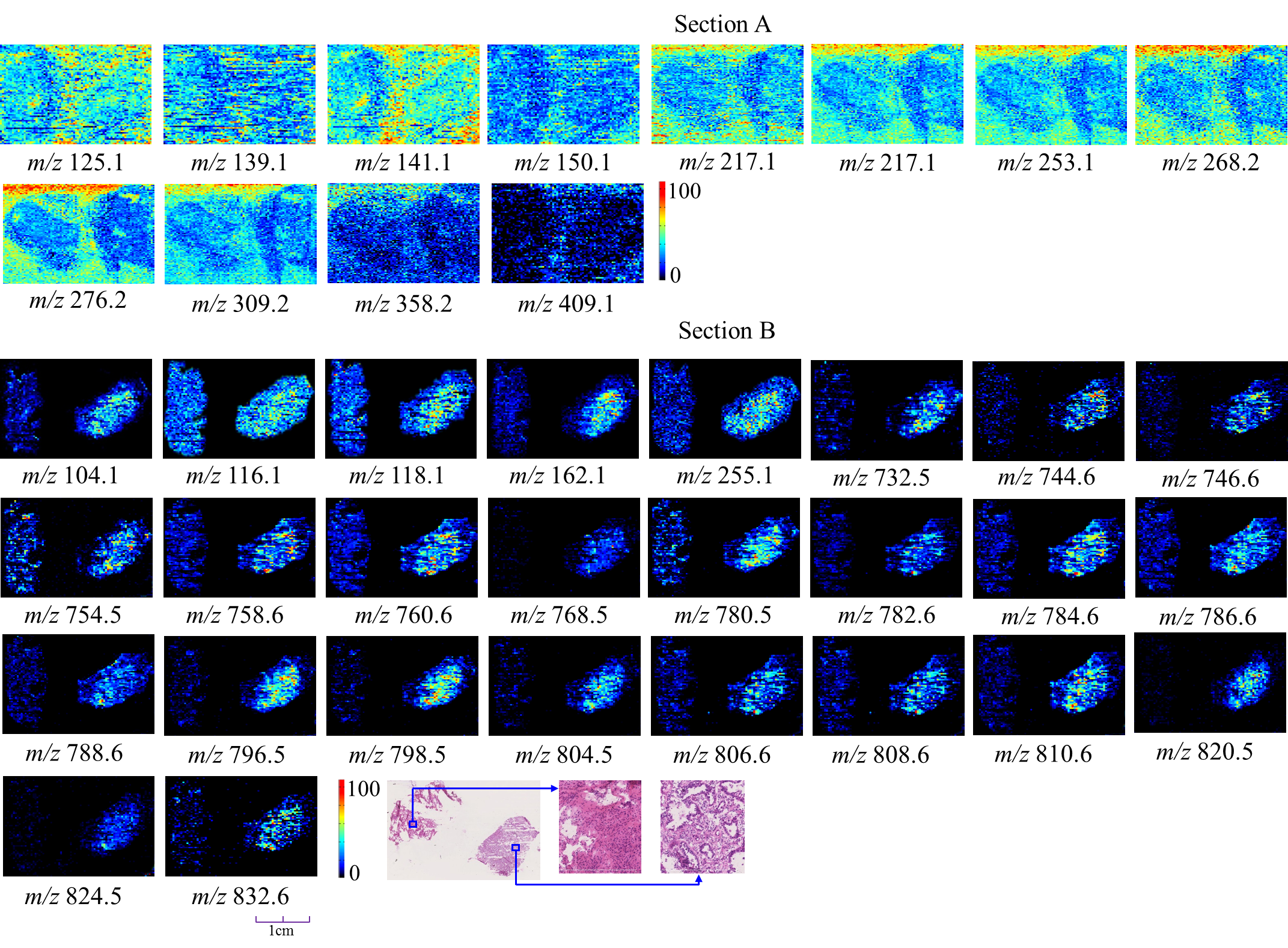


**Figure S3.** Imaging test results of the 38 discriminated variables. The 12 discriminated variables shown in section A were excluded due to their non-distinct contours, whereas the 26 discriminated variables shown in section B were kept due to their clear outline and consonance with the optical image of the H&E-stained section, the primary assignment results of these species was shown in Table S2.

**Table S2.** Potential biomarkers of lung tumors and their tentative identiﬁcations in positive-ion mode. (P < 0.001,VIP >1.5)

| No. | *m/z* | Postulated elemental composition | ppm Error | VIP value  (trend) | Potential results | Figure index |
| --- | --- | --- | --- | --- | --- | --- |
| 1 | 104.1067 | C_5_H_14_NO^+^ | ˗1.831 | 14.0 ↑ | Choline^[a]^ | Fig. S7-a |
| 2 | 116.0709 | C_5_H_10_NO_2_^+^ | 2.5406 | 2.01 ↑ | Proline ^[a]^ | Fig. S7-b |
| 3 | 118.0862 | C_5_H_12_NO_2_^+^ | ˗0.467 | 2.55 ↑ | Betaine ^[a]^ | Fig. S7-c |
| 4 | 162.1113 | C_7_H_16_NO_3_^+^ | ˗2.282 | 3.72 ↑ | L-carnitine^[a]^ | Fig. S7-d |
| 5 | 255.0910 | C_10_H_15_N_4_O_2_S^+^ | -0.090 | 1.89 ↑ | Unidentified | Fig. S7-e |
| 6 | 732.5535 | C_40_H_79_NO_8_P^+^ | ˗0.384 | 7.32 ↑ | PC(32:1)^[b]^ | Fig. S7-f |
| 7 | 744.5914 | C_42_H_83_NO_7_P^+^ | 1.656 | 3.37 ↑ | PC(P-34:1) | Fig. S7-g |
| 8 | 746.6058 | C_42_H_85_NO_7_P^+^ | ˗0.023 | 5.11 ↑ | PC(O-34:1) | Fig. S7-h |
| 9 | 754.5375 | C_40_H_78_NO_8_PNa^+^ | 2.351 | 5.06 ↑ | [PC(32:1)+Na]^+^ | Fig. S7-i |
| 10 | 758.5699 | C_42_H_81_NO_8_P^+^ | 0.614 | 8.58 ↑ | PC(16:0/18:2) | Fig. S7-j |
| 11 | 760.5850 | C_42_H_83_NO_8_P^+^ | 0.0206 | 14.08 ↑ | PC(16:0/18:1) | Fig. S7-k |
| 12 | 768.5853 | C_42_H_84_NO_7_PNa^+^ | -3.203 | 5.74 ↑ | [PC(O-16:0/18:1)+Na]^+^ | Fig. S7-l |
| 13 | 780.5509 | C_42_H_80_NO_8_PNa^+^ | ˗0.6134 | 6.35 ↑ | [PC(16:0/18:2)+Na]^+^ | Fig. S7-m |
| 14 | 782.5695 | C_42_H_82_NO_8_PNa^+^ | 0.733 | 13.36 ↑ | [PC(16:0/18:1)+Na]^+^ | Fig. S7-n |
| 15 | 784.5832 | C_42_H_84_NO_8_PNa^+^ | 0.668 | 8.94 ↑ | [PC(34:0)+Na]^+^ | Fig. S7-o |
| 16 | 786.6022 | C_44_H_85_NO_8_P^+^ | 1.8632 | 8.83 ↑ | PC(18:0/18:2) | Fig. S7-p |
| 17 | 788.6147 | C_44_H_87_NO_8_P^+^ | ˗2.0091 | 6.47 ↑ | PC(36:1) | Fig. S7-q |
| 18 | 796.6155 | C_44_H_88_NO_7_PNa^+^ | -4.471 | 6.78 ↑ | [PC(P-18:0/18:0)+Na]^+^ | Fig. S7-r |
| 19 | 798.5430 | C_42_H_82_NO_8_PK^+^ | ˗1.4615 | 9.24 ↑ | [PC(34:1)+K]^+^ | Fig. S7-s |
| 20 | 804.5521 | C_44_H_80_NO_8_PNa^+^ | 0.8963 | 6.23 ↑ | [PC(16:0/20:4)+Na]^+^ | Fig. S7-t |
| 21 | 806.5667 | C_44_H_82_NO_8_NaP^+^ | ˗1.2756 | 7.05 ↑ | [PC(36:3)+Na]^+^ | Fig. S7-u |
| 22 | 808.5854 | C_44_H_84_NO_8_PNa^+^ | 1.3863 | 8.21 ↑ | [PC(18:1/18:1)+Na]^+^ | Fig. S7-v |
| 23 | 810.5997 | C_44_H_86_NO_8_PNa^+^ | ˗1.276 | 8.36 ↑ | [PC(18:0/18:1)+Na]^+^ | Fig. S7-w |
| 24 | 820.6174 | C_46_H_88_NO_7_PNa^+^ | ˗1.662 | 5.13 ↑ | [PC(38:2)+Na]^+^ | Fig. S7-x |
| 25 | 824.5574 | - | - | 5.53 ↑ | Unidentified | - |
| 26 | 832.5839 | C_46_H_84_NO_8_PNa^+^ | 1.4708 | 5.89 ↑ | [PC(18:1/20:3)+Na]^+^ | Fig. S7-y |
| [a] Indicates that the metabolites were confirmed with authentic standards.  [b] (X:Y) represents the number of carbon atoms (X) and the number of double bonds (Y) in the fatty acid chains. PC: glycerophosphocholine. | | | | | | |


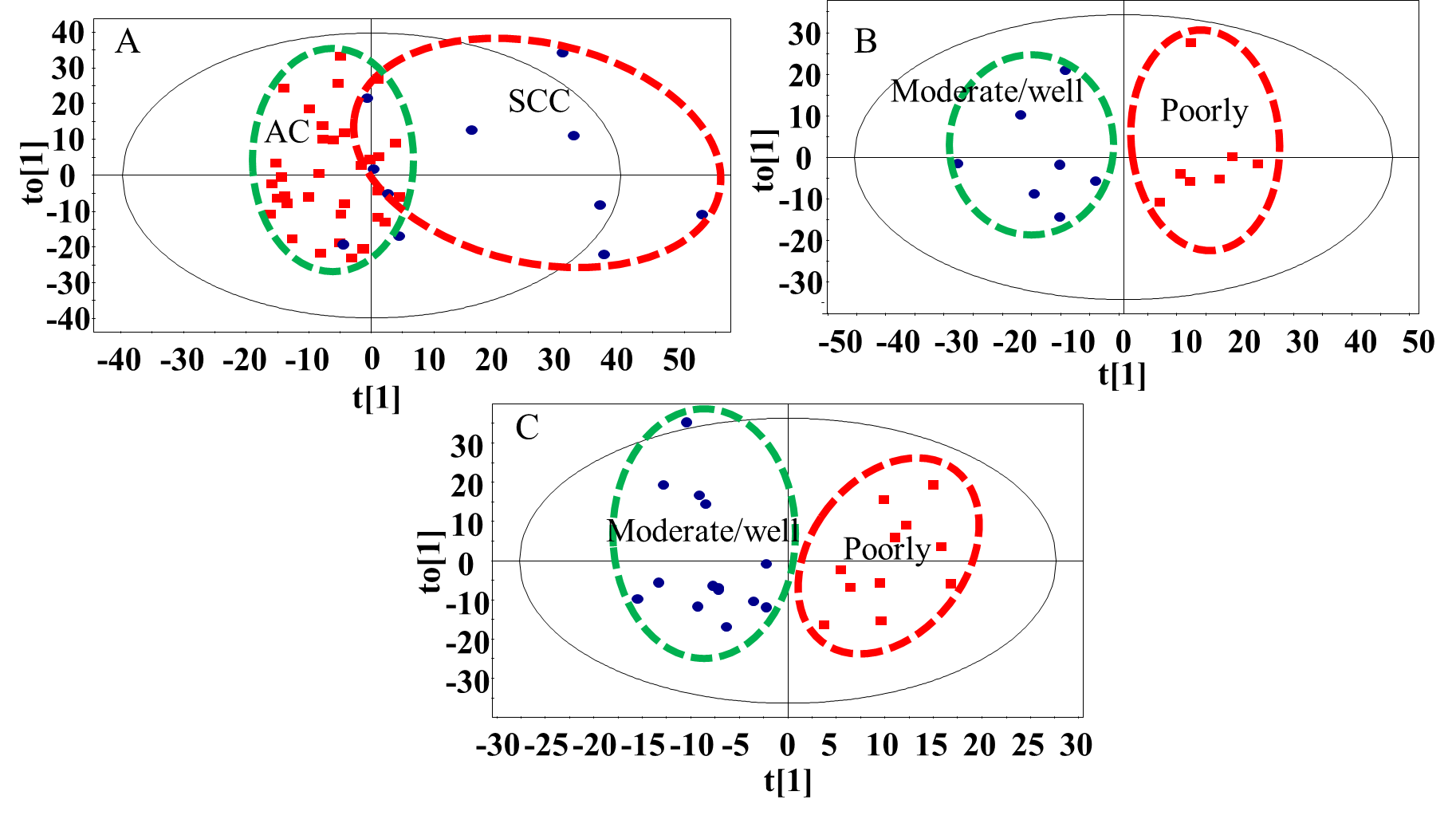


**Figure S4.** OPLS-DA models for discrimination of subtype and differentiation degree of lung cancer. A) Pathological type (AC vs. SCC), B) Differentiation degree of SCC (poorly-differentiated and moderate/well-differentiated), C) Differentiation degree of AC (poorly-differentiated and moderate/well-differentiated).

**Table S3.** Potential biomarkers that discriminate SCC and AC, with their tentative assignment results. (P < 0.001,VIP >1.5)

| No. | *m/z* | Postulated elemental composition | ppm Error | VIP value | Potential results | Figure index |
| --- | --- | --- | --- | --- | --- | --- |
| 1 | 269.1235 | C_11_H_17_N_4_O_4_^+^ | ˗3.461 | 2.79 | Hydroxyprolyl-Histidine | Fig. S8-a |
| 2 | 297.1542 | C_13_H_21_N_4_O_4_^+^ | ˗5.154 | 2.80 | Unidentified | Fig. S8-b |
| 3 | 429.2403 | - | - | 2.16 | Unidentified | - |

**Table S4.** Potential biomarkers that discriminate differentiation degree (poorly- and moderate/well-differentiated) for AC, with their tentative assignment results. (P < 0.05,VIP > 2)

| No. | *m/z* | Postulated elemental composition | ppm Error | VIP value | Potential results | Figure index |
| --- | --- | --- | --- | --- | --- | --- |
| 1 | 703.5764 | C_39_H_80_O_6_PN_2_^+^ | 2.201 | 2.04 | SM(34:1)^[a]^ | Fig. S9-a |
| 2 | 734.5698 | C_40_H_81_NO_8_P^+^ | 0.502 | 3.63 | PC(16:0/16:0) | Fig. S9-b |
| 3 | 758.5699 | C_42_H_81_NO_8_P^+^ | 0.618 | 4.96 | PC(16:0/18:2) | Fig. S7-j |

[a] (X:Y) represents the number of carbon atoms (X) and the number of double bonds (Y) in the fatty acid chains. PC: glycerophosphocholine, SM: sphingomyelin.


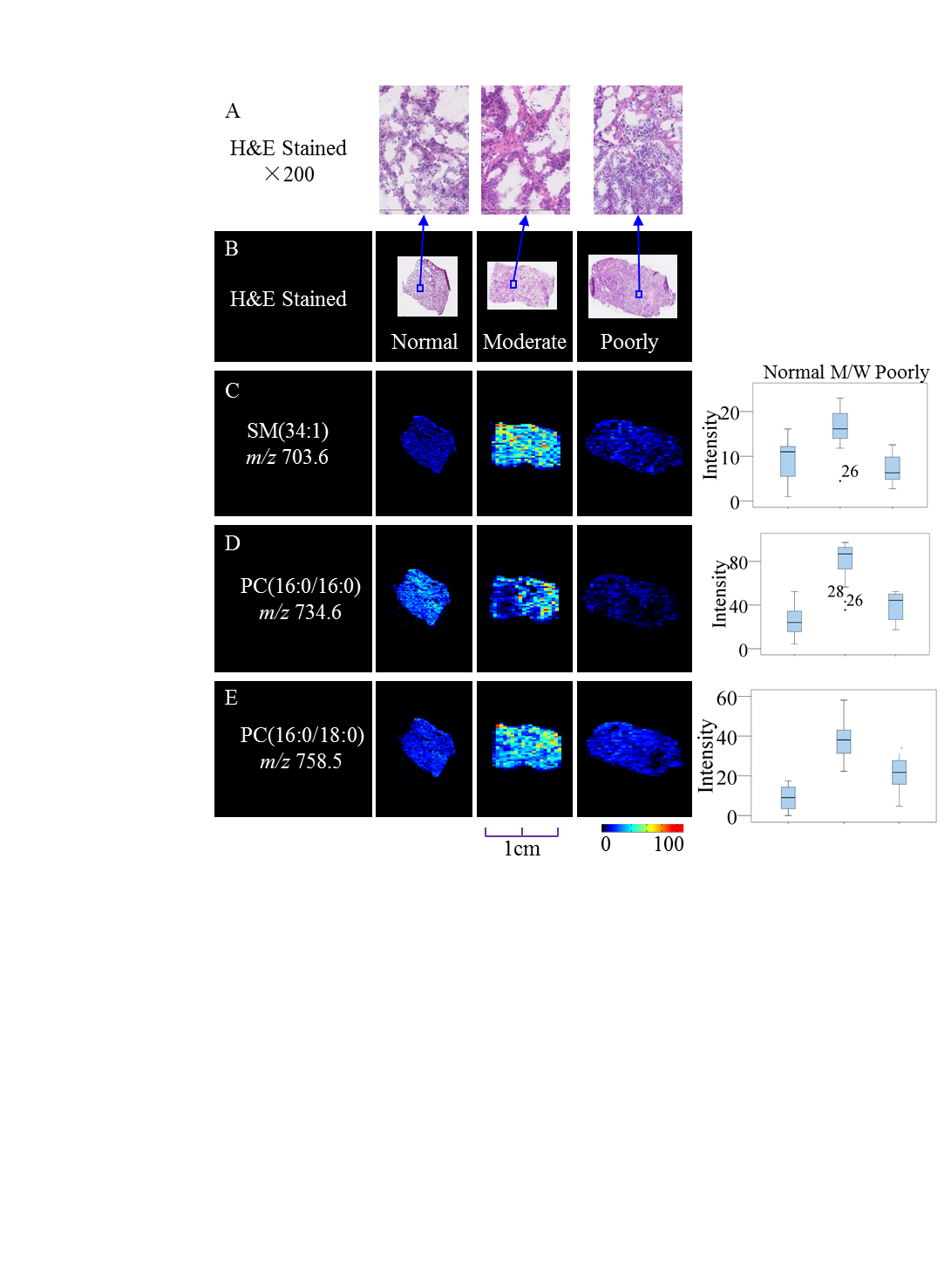


**Figure S5.** Distribution of representative potential biomarkers across tissue sections from AC, with degree of differentiation in the tumorous and normal tissue. A-B) Optical images of the corresponding H&E-stained sections and the amplified figures (×200). C–E) Ion images and corresponding statistical box plots. M/W: moderate/well. Column 1: potential biomarkers with their molecular ions (*m/z*); Column 2: ion images from adjacent normal tissue sections; Column 3: ion images from tumorous tissue sections with moderate differentiation; Column 4: ion images from tumorous tissue sections with poorly differentiation; Column 5: the statistical box plots shows the ion intensity of the on normal and tumorous tissue sections with the degree of differentiation.


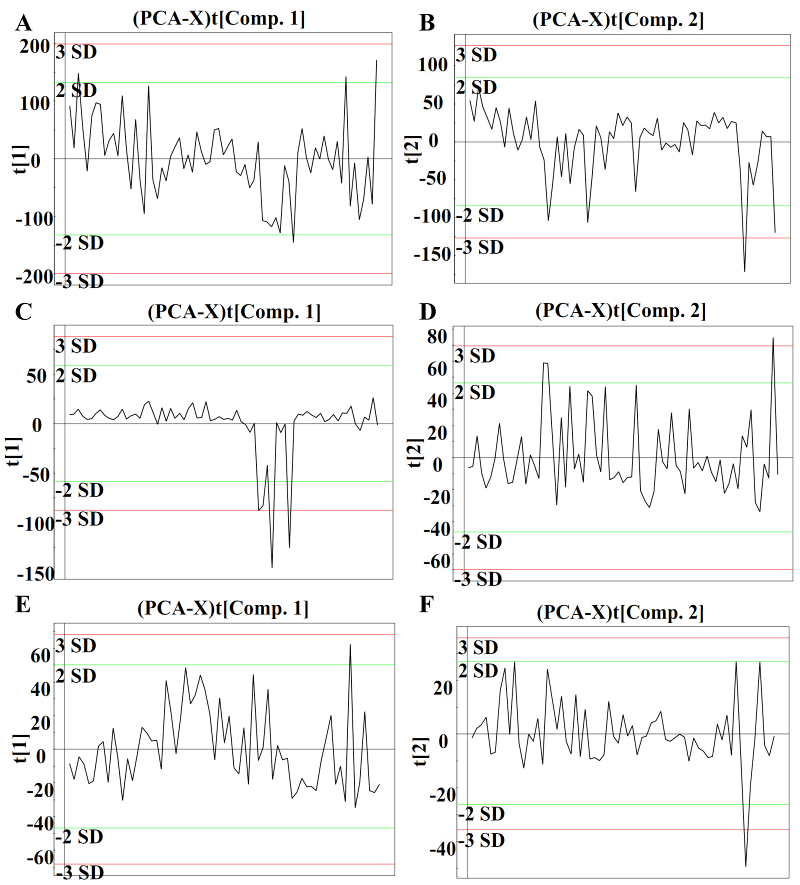


**Figure S6.** Line plots of the data extracted from a blank area generated by PCA using component 1 and 2 shows the stability of AFAI-MSI system. AFAI-MSI analysis deviation was evaluated by the distribution of the runs. X-axis: run order; Y-axis: standard deviation. Line plots depict the first and second components from positive ion mode MSI over mass range 100–500 Da (A, B) and 500–1000 Da (C, D), and from negative ion mode MSI over mass range 100–500 Da (E, F).


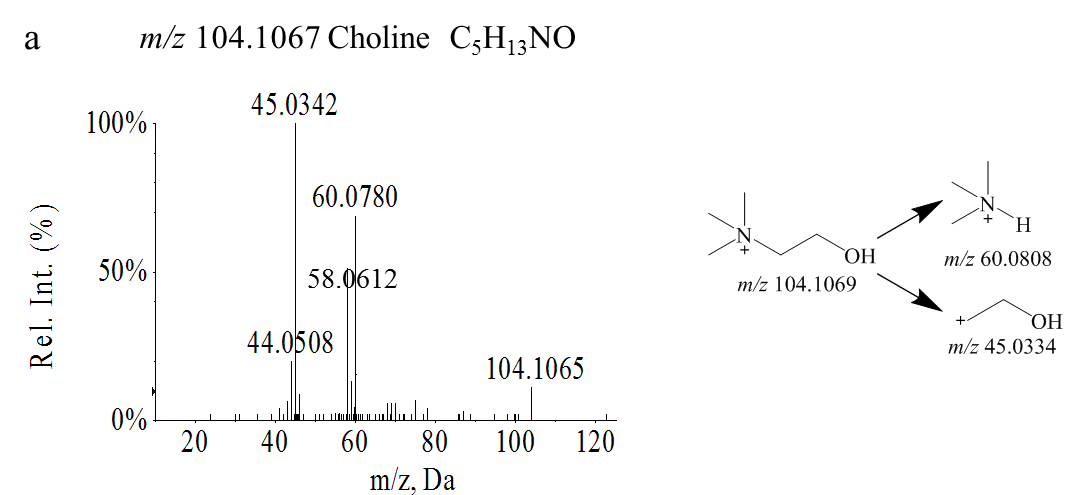


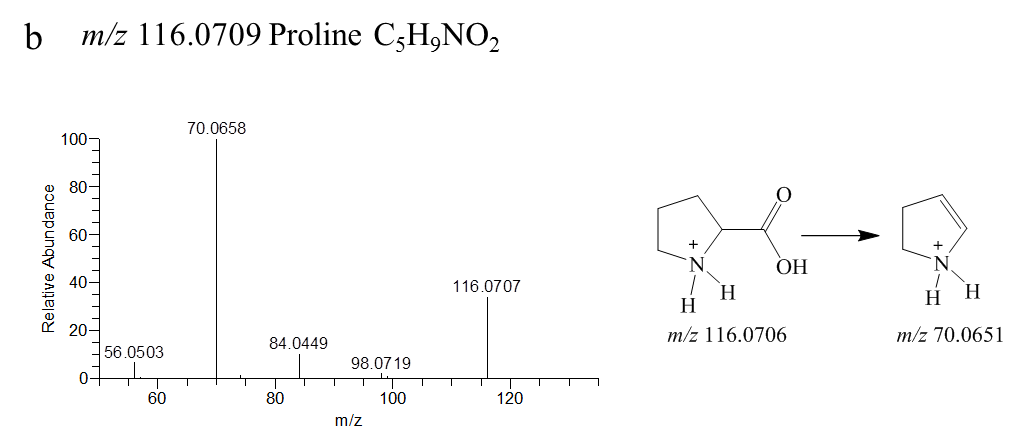


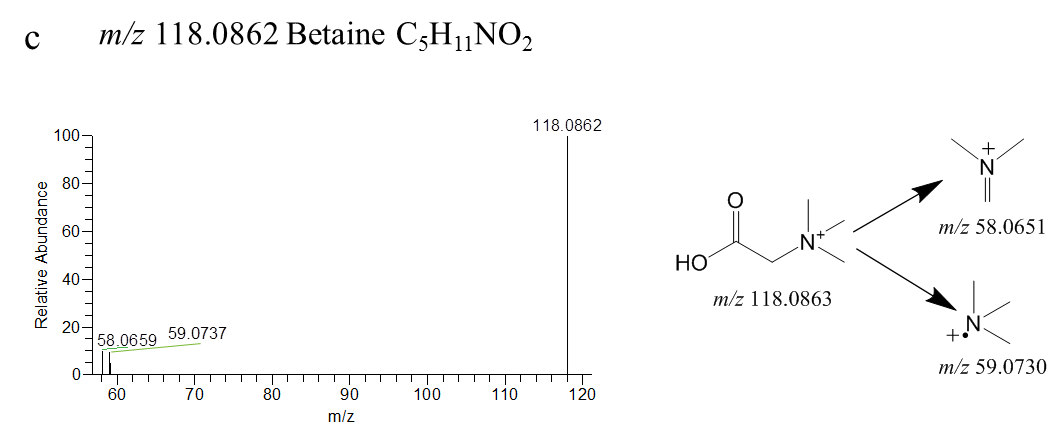


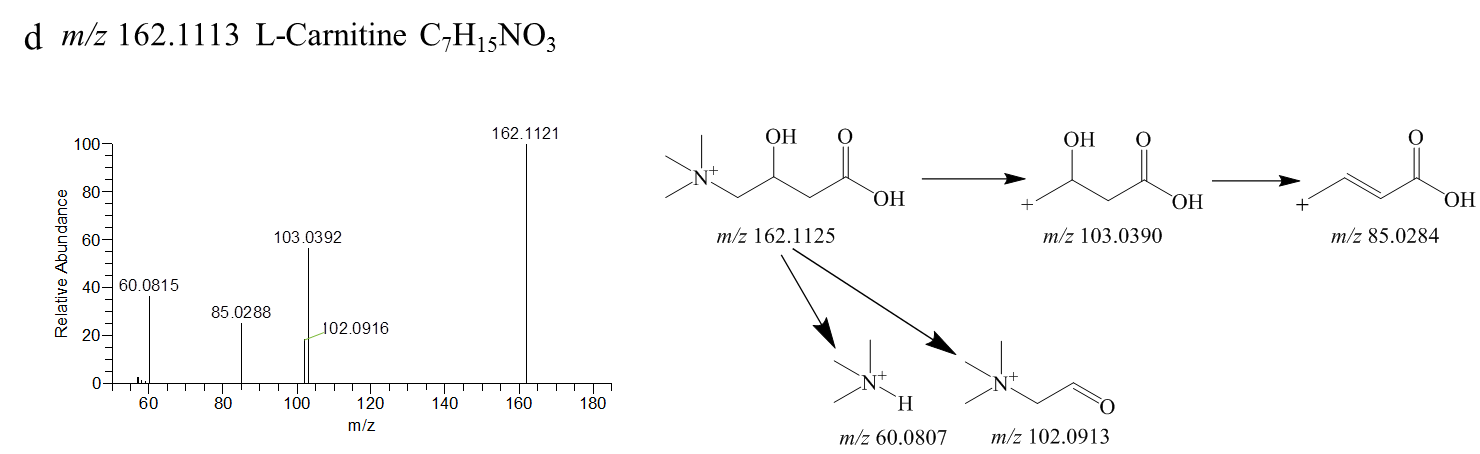


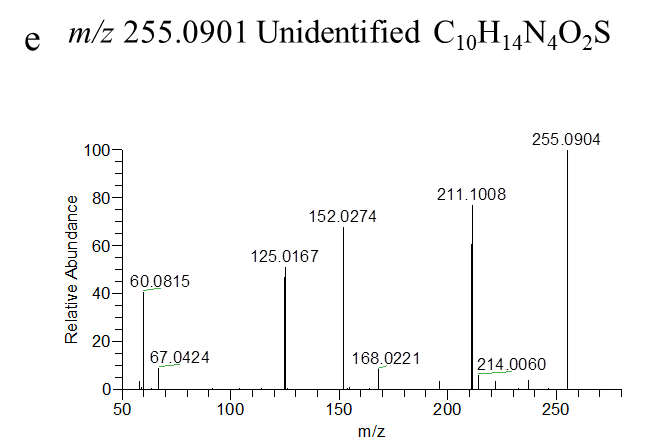


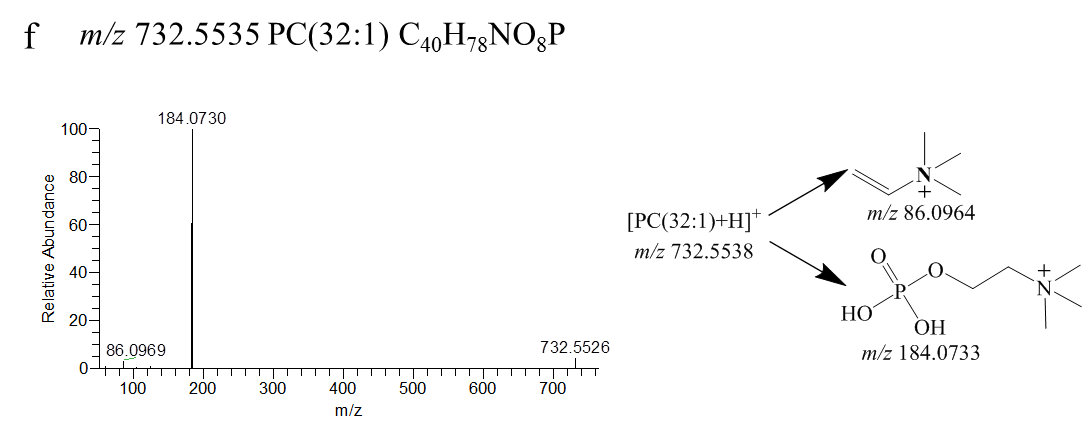


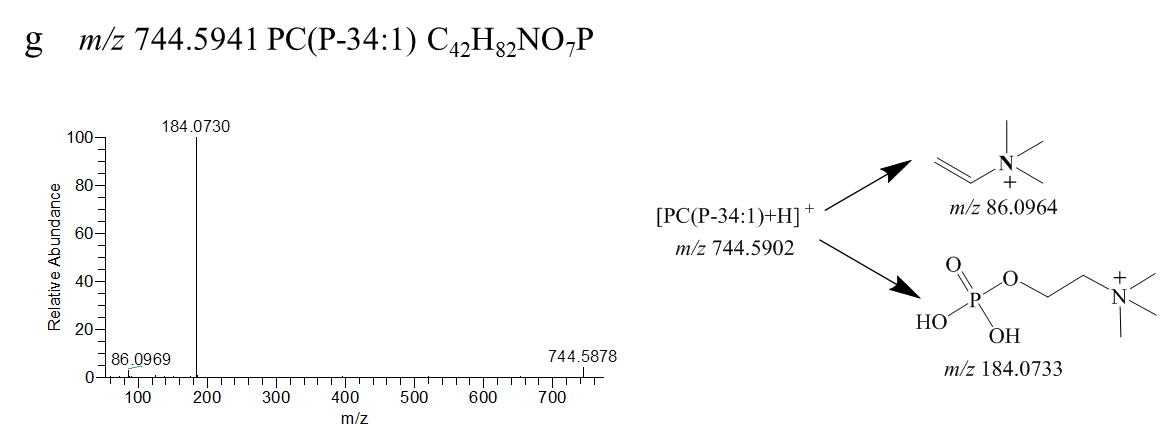


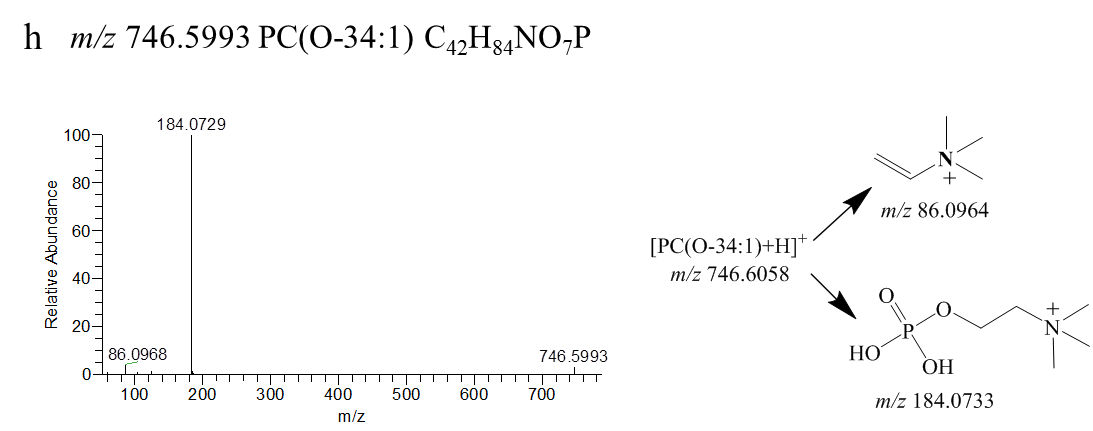


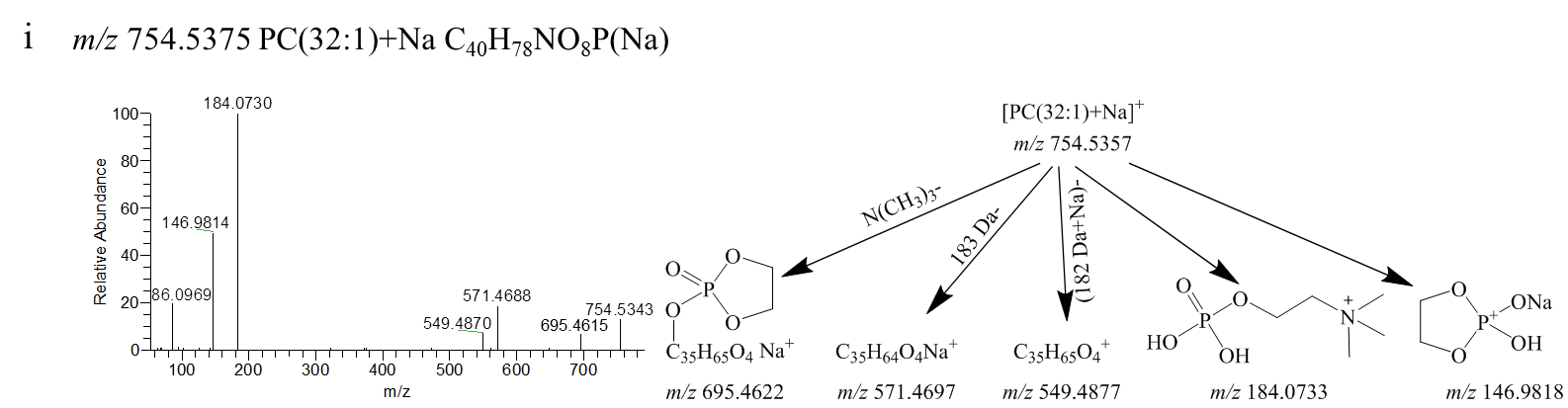


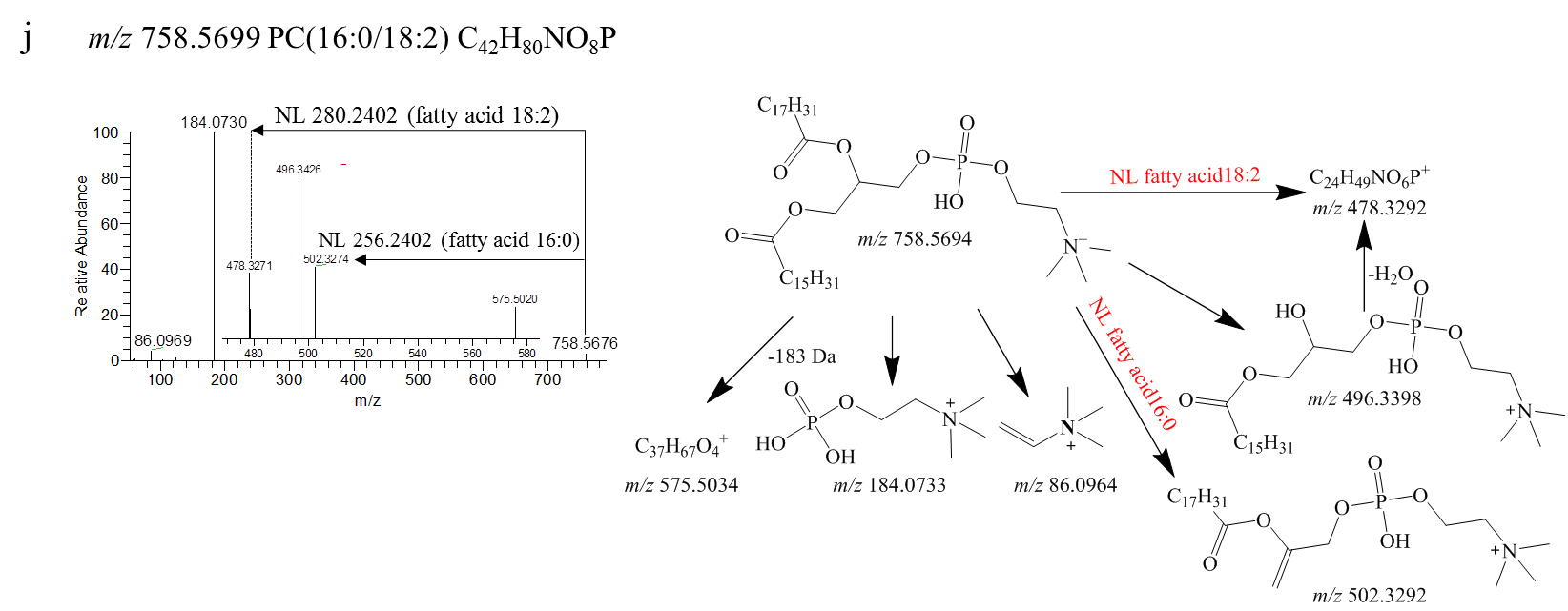


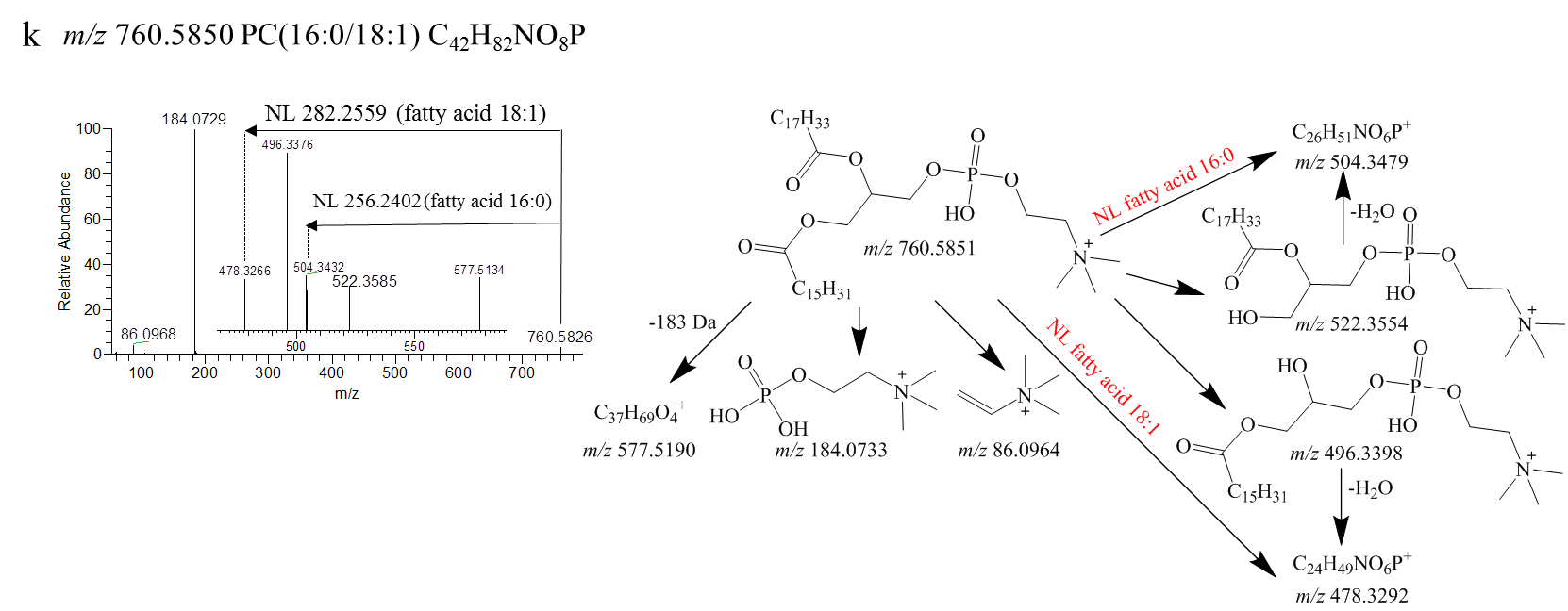


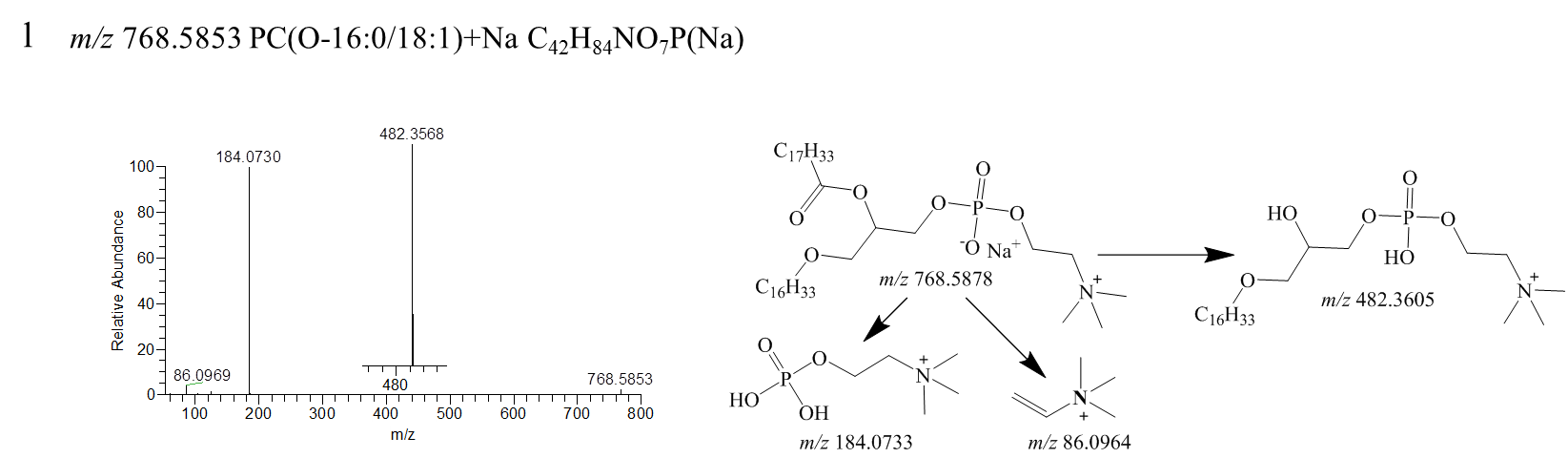


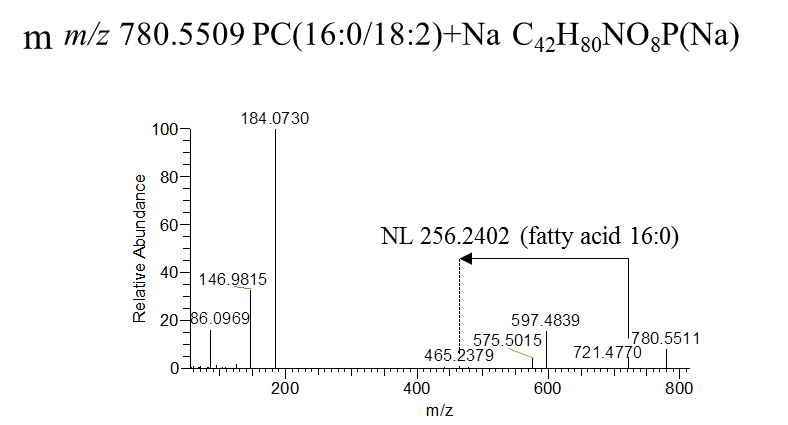


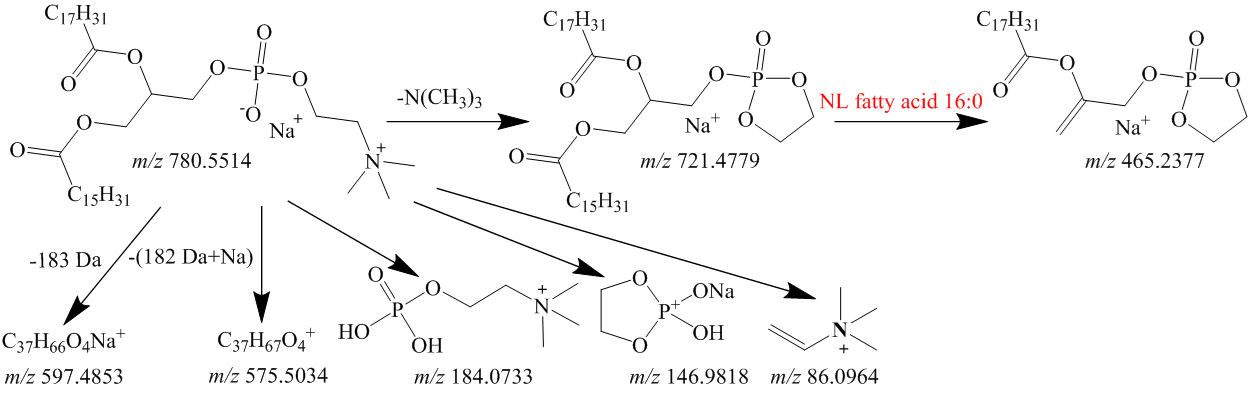


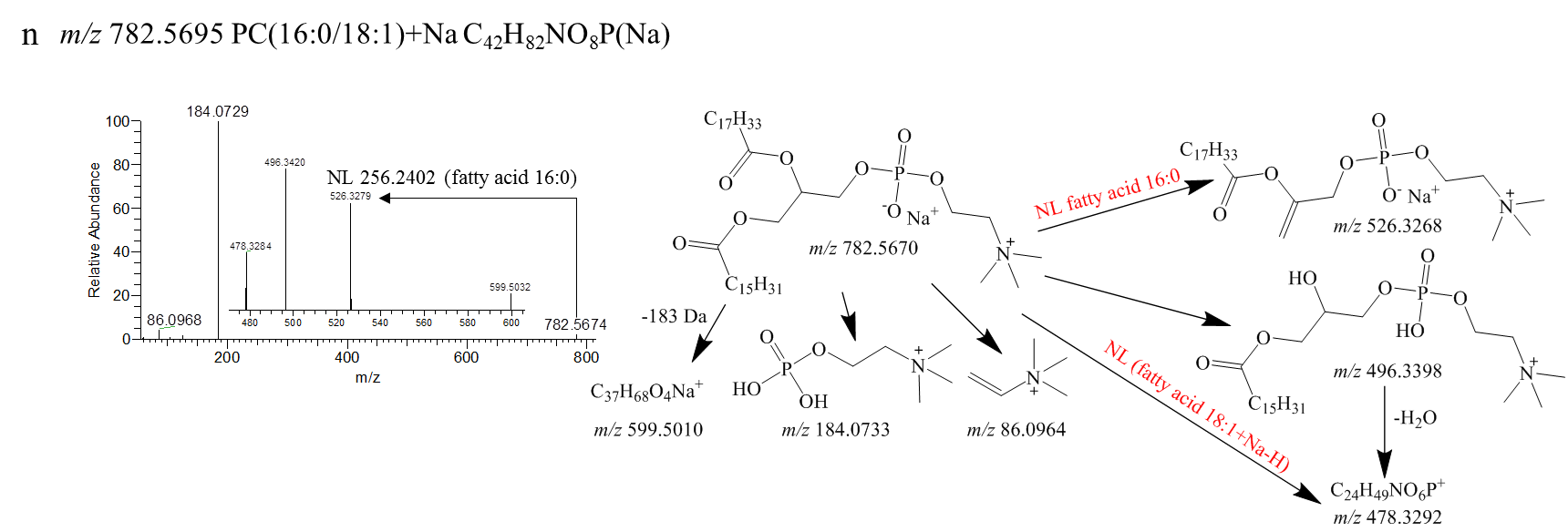


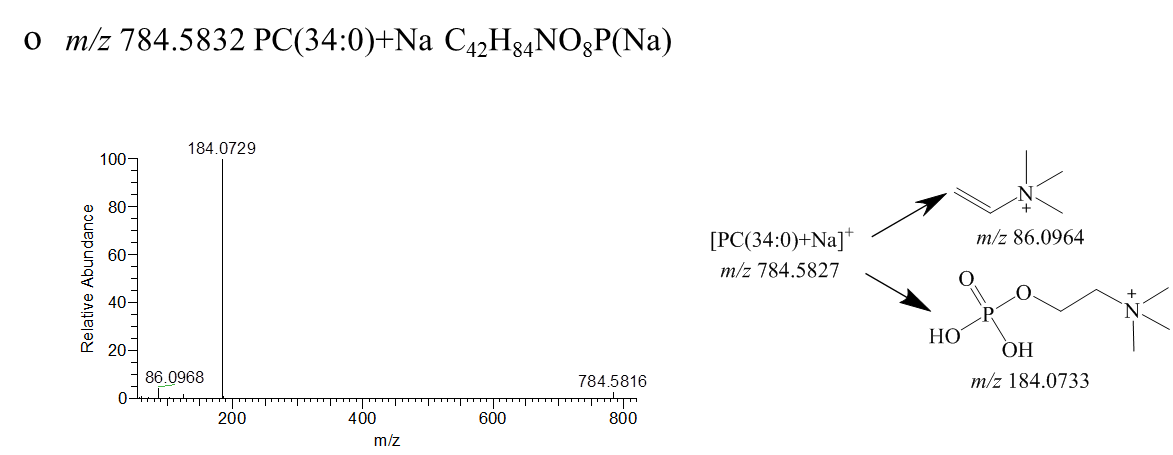


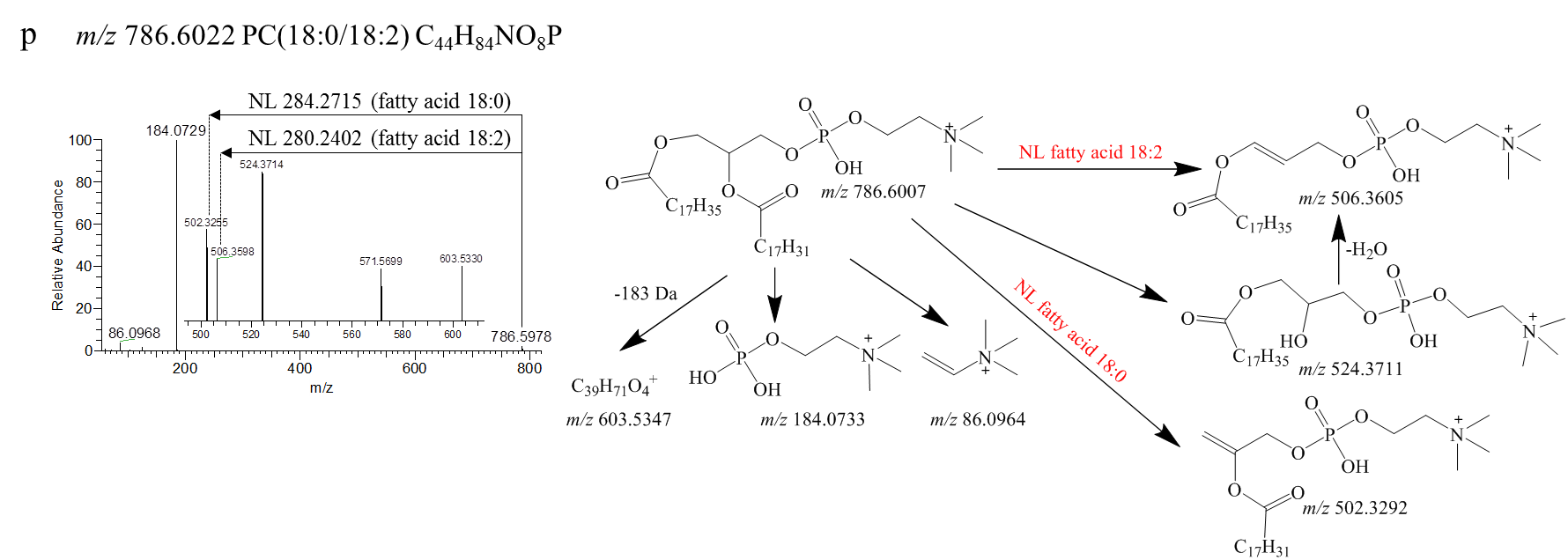


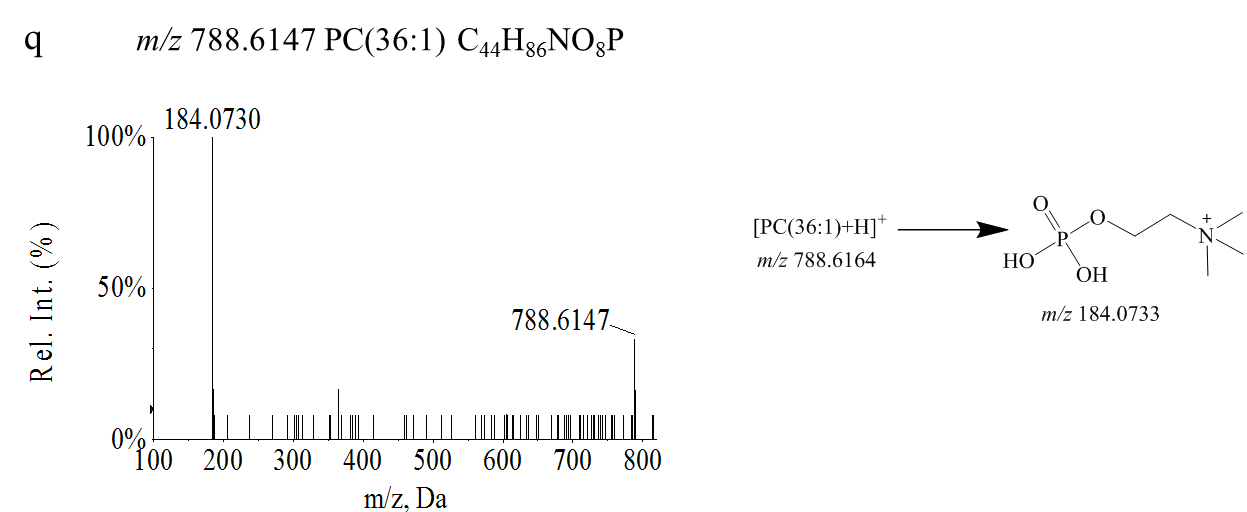


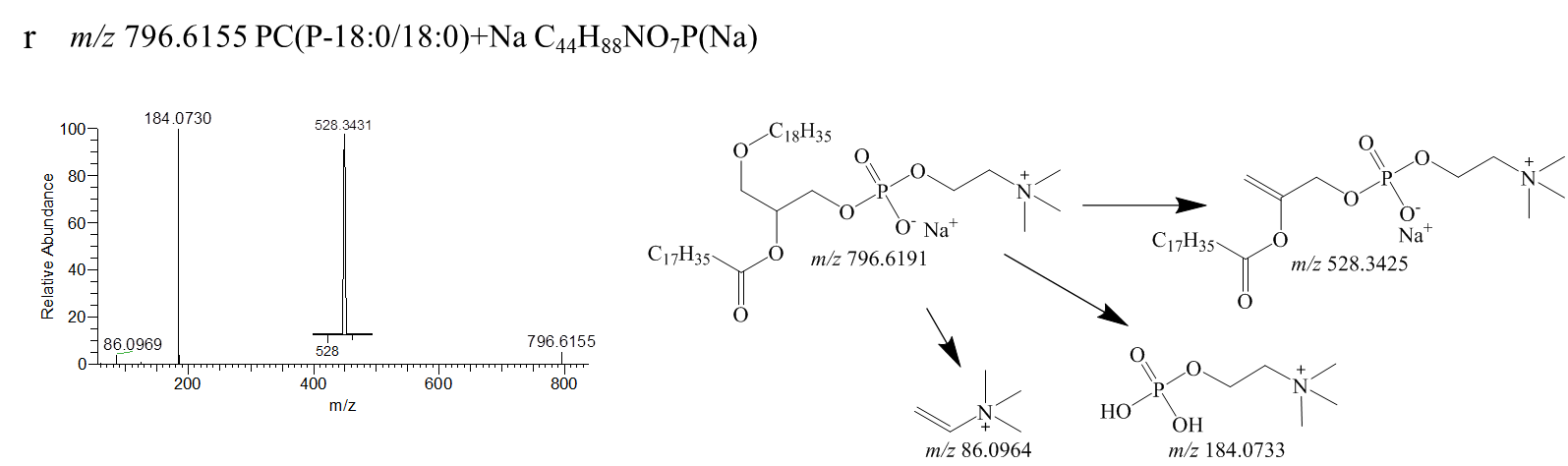


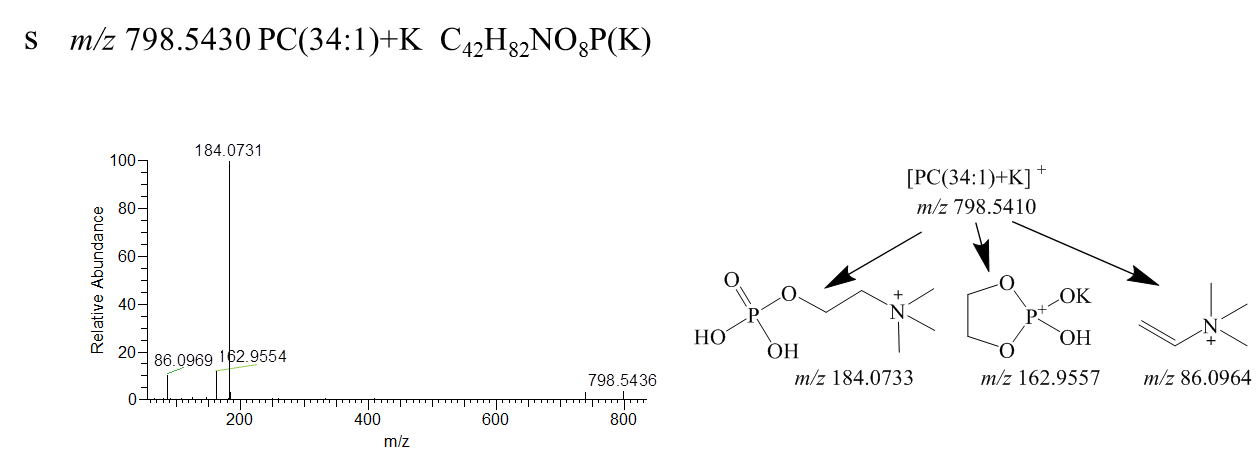


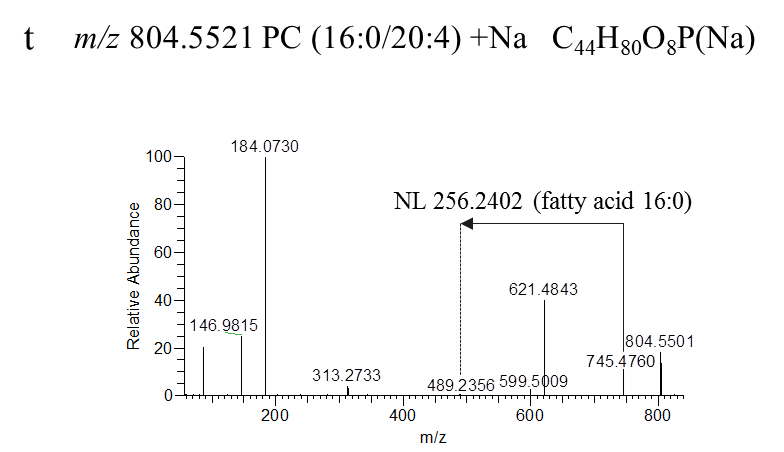


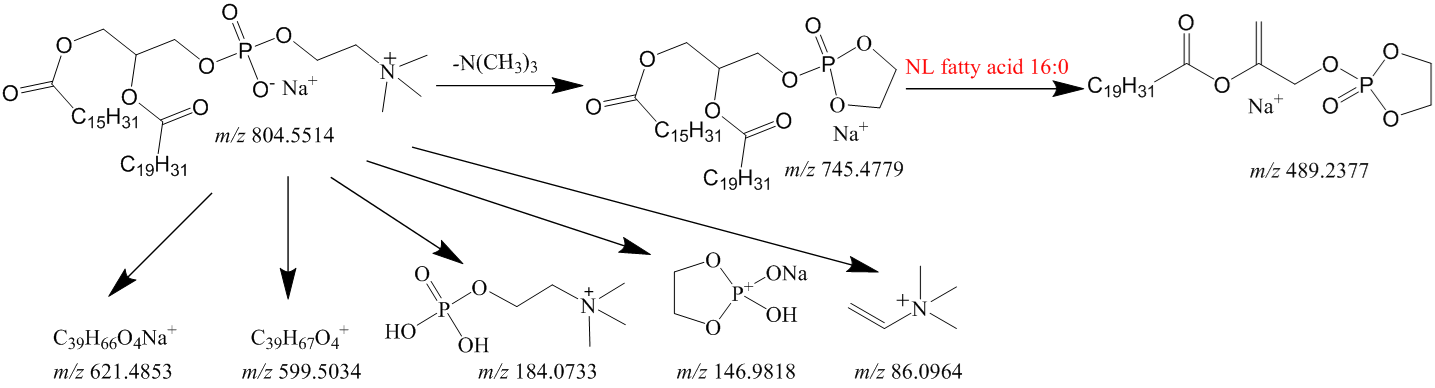


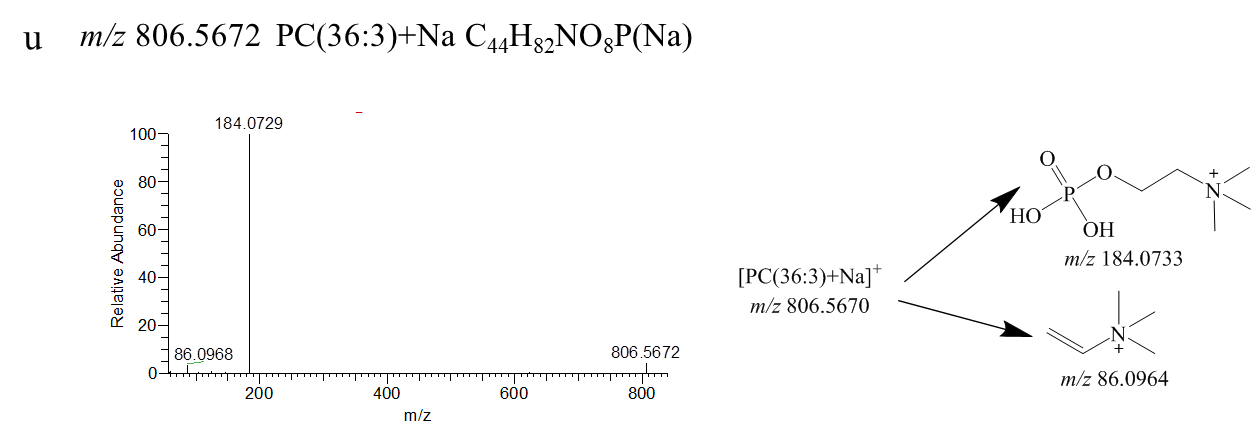


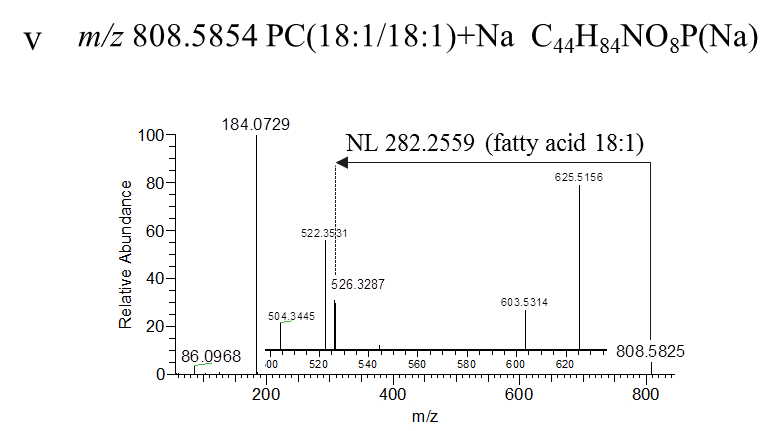


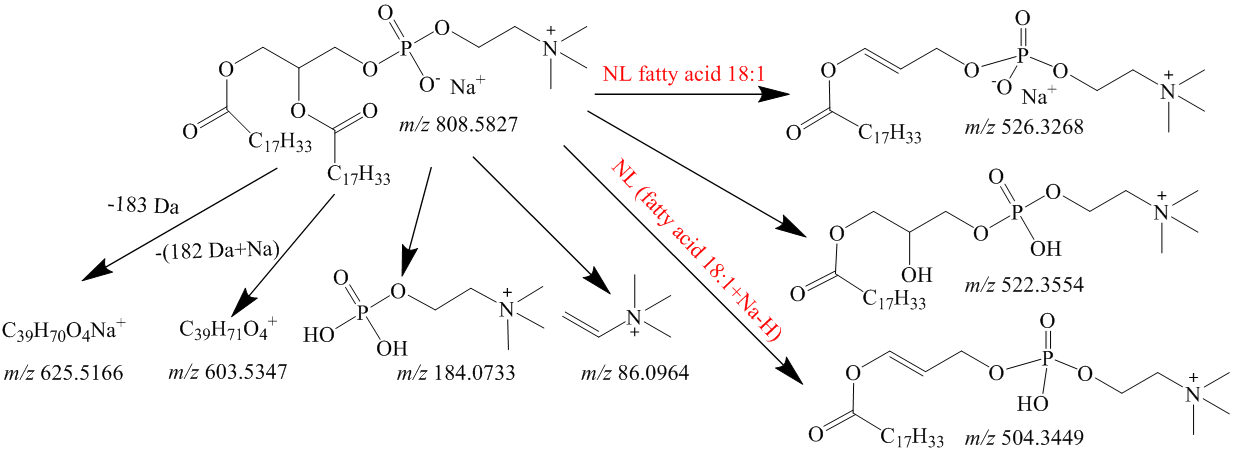


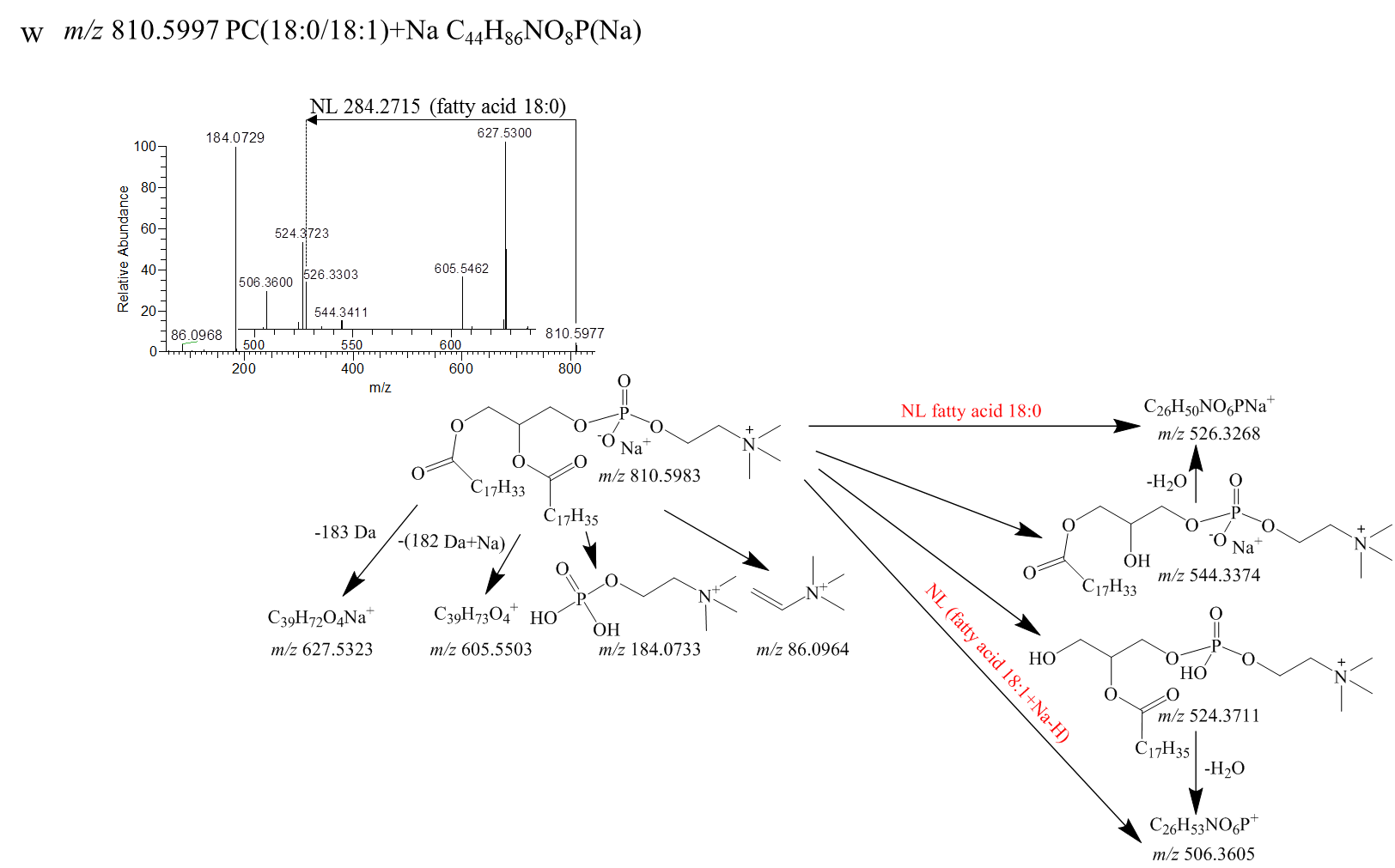


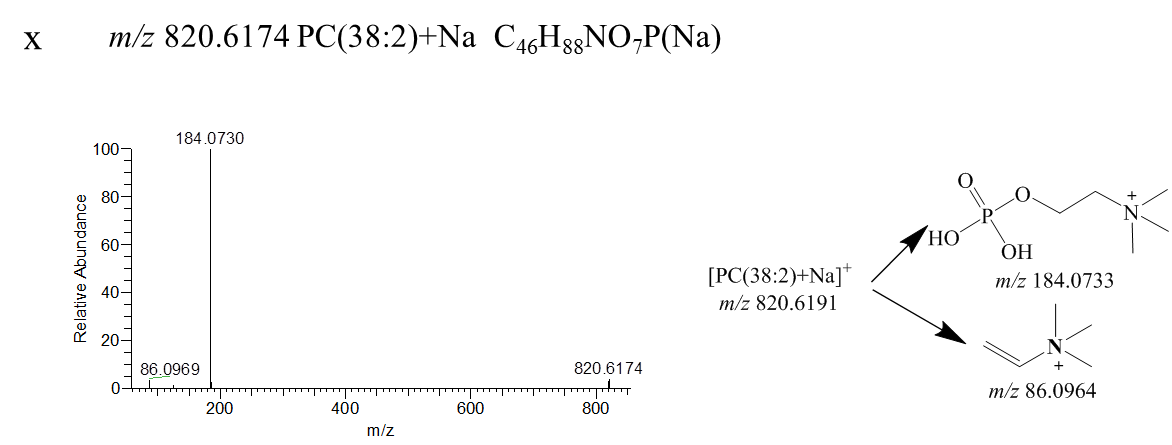


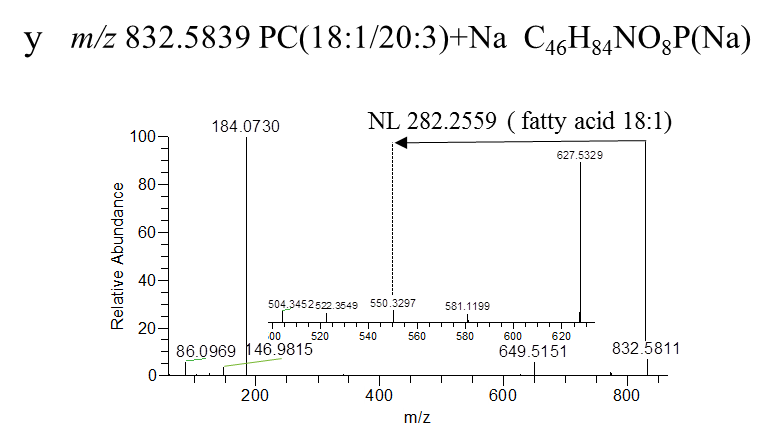


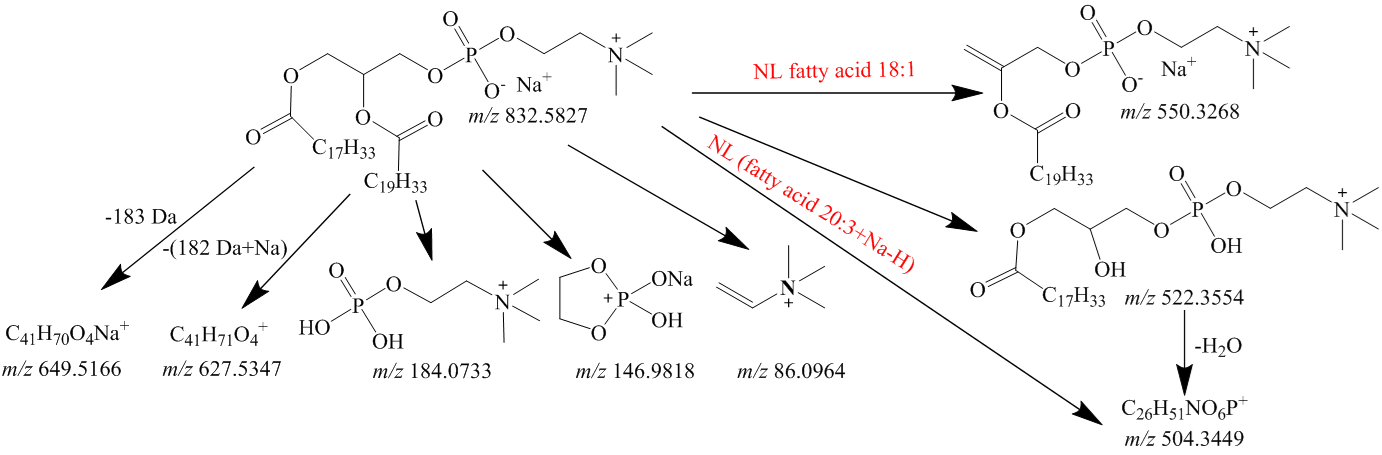


**Figure S7.** MS/MS spectra and the proposed dissociation pathways for the discriminated metabolites identification (Tumor VS non-tumor).


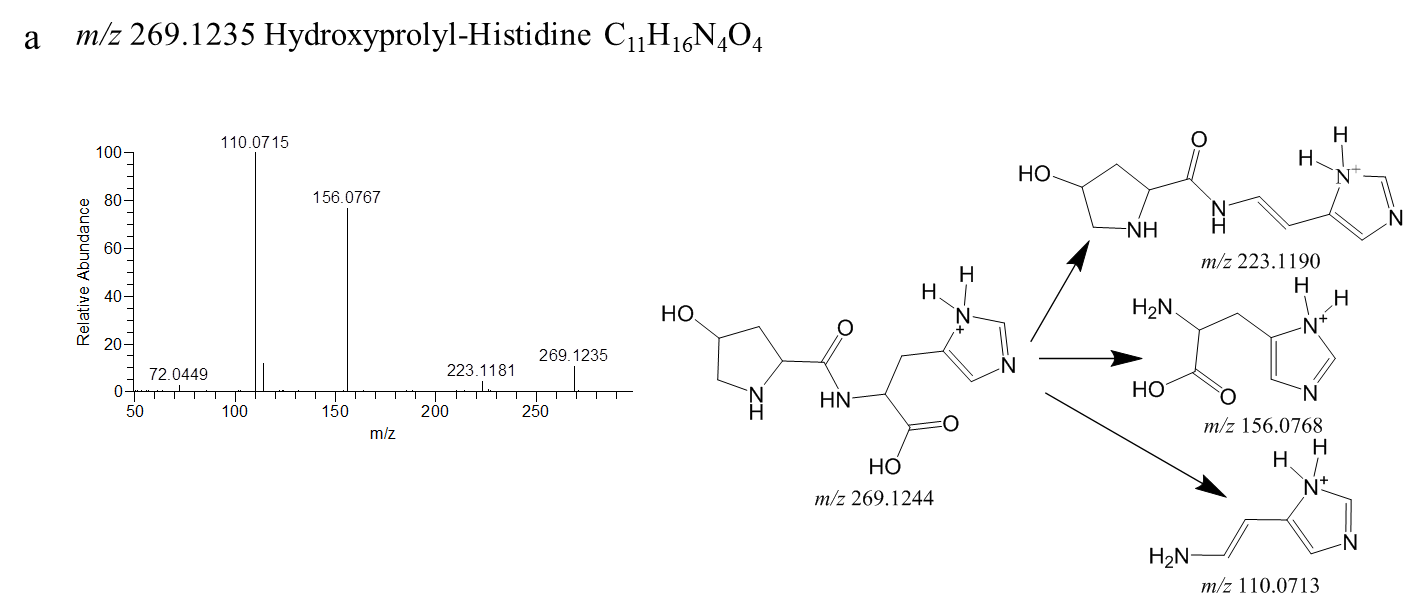


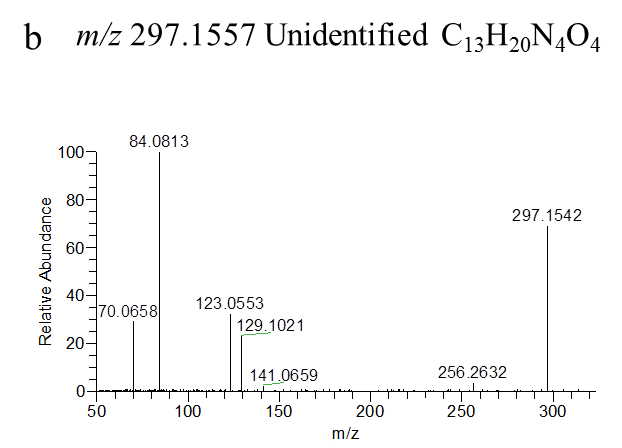


**Figure S8.**  MS/MS spectra and the proposed dissociation pathways for the discriminated metabolites identification (AC VS SCC).


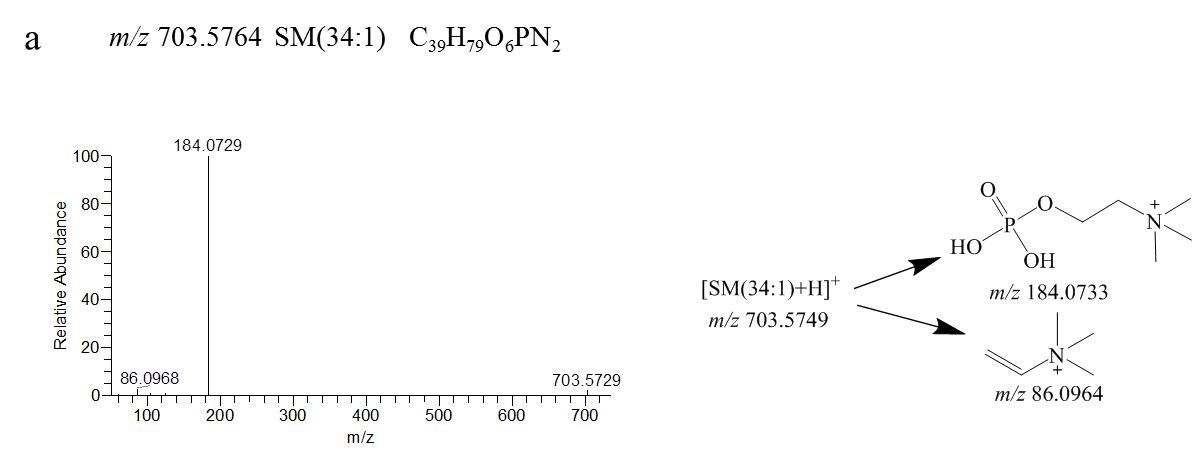


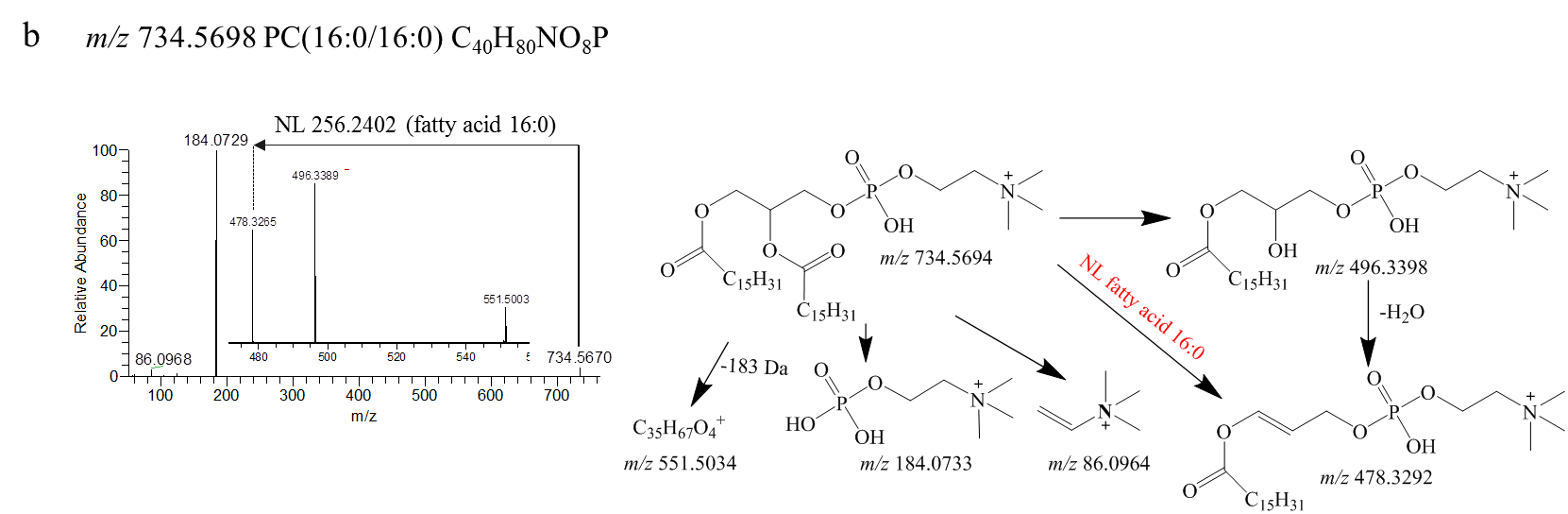


**Figure S9.** MS/MS spectra and the proposed dissociation pathways for the discriminated metabolites identification (Differentiation degree for AC).
